# Robust design of bicycle infrastructure networks

**DOI:** 10.1038/s41598-025-99976-9

**Published:** 2025-05-03

**Authors:** Christoph Steinacker, Mads Paulsen, Malte Schröder, Jeppe Rich

**Affiliations:** 1 Chair of Network Dynamics, Center for Advancing Electronics Dresden (cfaed) and Institute of Theoretical Physics, TUD Dresden University of Technology, 01062 Dresden, Germany; 2 Transportation Science Division, Department of Technology, Management and Economics, Technical University of Denmark, 2800 Kgs. Lyngby, Denmark

**Keywords:** Complex networks, Computational science, Civil engineering

## Abstract

Promoting active mobility like cycling relies on the availability of well-connected, high-quality bicycle networks. However, expanding these networks over an extended planning horizon presents one of the most complex challenges in transport science. This complexity arises from the intricate interactions between infrastructure availability and usage, such as network spillover effects and mode choice substitutions. In this paper, we approach the problem from two perspectives: direct optimization methods, which generate near-optimal solutions using operations research techniques, and conceptual heuristics, which offer intuitive and scalable algorithms grounded in network science. Specifically, we compare direct welfare optimization with an inverse network percolation approach to planning cycle superhighway extensions in Copenhagen. Interestingly, while the more complex optimization models yield better overall welfare results, the improvements over simpler methods are small. More importantly, we demonstrate that the increased complexity of planning approaches generally makes them more vulnerable to input uncertainty, reflecting the bias-variance tradeoff. This issue is particularly relevant in the context of long-term planning, where conditions change during the implementation of the planned infrastructure expansions. Therefore, while planning bicycle infrastructure is important and renders exceptionally high benefit-cost ratios, considerations of robustness and ease of implementation may justify the use of more straightforward network-based methods.

## Introduction

Promoting active mobility, like walking and cycling, is important for achieving sustainable mobility. However, this is only possible with a sufficiently developed infrastructure. Specifically for cycling, demand strongly relies on the quality and quantity of the available bicycle infrastructure^1–3^. Due to its low costs and space requirements compared to other modes of transport^4,5^, there is growing evidence of large, positive welfare benefits of bicycle infrastructure for society^6–11^.

**Fig. 1:**
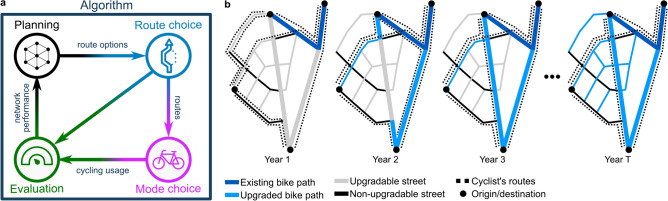
The network expansion problem. (**a**) Designing efficient infrastructure network expansions is a complex problem due to interactions and feedback effects between the available and planned infrastructure, the possible routing options, the resulting cycling demand, and the evaluation of the network quality. (**b**) Sketch of bike path network extensions over time (left to right). Cyclists prefer to travel along bike paths (blue lines) but avoid large streets without bike paths (thick gray lines). As new bike paths are added (light blue), the cyclists change their routes (black dotted lines) to use the new routing options. This, in turn, affects the importance of all other existing and potential bike paths in the network, and thus changes which bike paths should be constructed next.

However, designing efficient network expansions over time represents one of the most challenging problems in urban planning^12,13^. The complexity of these network design or network expansion problems^14,15^ stems from their dynamic nature, where investments in one period affect the usage of the network and the benefits of additional investments in the future (Fig. 1). These feedback effects include changes in the routes of cyclists and spillovers from other transport modes as cycling becomes faster and more convenient. The problem presents itself as a dynamic optimization problem with a structure similar to the one presented in the seminal work of Bellman^16^. Compared to complex network design problems for multimodal or congested transport, bicycle infrastructure planning does not typically include direct interactions between individual travelers. Nonetheless, the problem remains highly complex^17^ due to the strong interactions and indirect feedback loops between available infrastructure, network quality, cycling demand, and route choice (Fig. 1) and cannot be solved without substantial simplifications of the underlying problem. This has resulted in a divide between two algorithmic approaches to addressing the problem.

One school addresses the problem from the classical operations research perspective, typically framing it as a mathematical program to optimize various objectives. These objectives can range from optimizing travel time^18^or distance and safety indicators^19^over minimizing cost and investments^20^to maximizing connectedness and directness of routes expressed in a set-covering mixed-integer linear programming (MILP)^21^. Many approaches combine multiple quality measures in a weighted multiple criteria function to represent the costs and benefits of all stakeholders involved^22,23^or employ Pareto-optimization to identify different feasible networks, for example based on the trade-off in travel times between bike and car traffic^24^. In recent years, a new class of operations research models has emerged, which more closely follows traditional cost-benefit analyses and aims to optimize a general welfare function expressed in terms of the net present value (NPV) of the infrastructure^7,8,25^. The net present value models also combine multiple criteria, all weighted into a common monetary base, to evaluate the total societal benefit of network extensions. In the reference model applied in this paper, the multi-criteria welfare function includes travel time benefits for cyclists, reduced healthcare costs for society due to the benefits of cycling, and the cost of investments and infrastructure maintenance^8^. Typically, to solve these problems on a large scale, various simplifications are required to enable the evaluation of the objective function or the optimization process itself, such as heuristic search of the possible extension pathways, aggregation and linearization techniques to simplify complex computations, or treating the optimization as a Markov chain problem and iteratively finding the next expansion steps based on the current network state instead of computing all expansion steps at once. Overall, the operations research perspective focuses more strongly on devising efficient solution methods and achieving network extension plans as close to optimal as possible.

The second school has grown out of the network science community and often approaches the problem more conceptually. These network science approaches analyze large-scale network data^26^and employ simplified heuristic models of network expansion based on conceptual quality measures including connecting fragmented components^27–29^, directness^30,31^, effective shortest path distances^32^ and coverage of the network^33,34^. A recent model taken as a reference in this paper, for example, generates a sequence of infrastructure networks by iteratively deconstructing a fully expanded network based on a flexible network evaluation scheme^32^. Overall, unlike the operations research perspective, network science-inspired approaches prioritize understanding common structural characteristics of efficient networks over identifying the exact optimal solution. This understanding can, for instance, inform heuristic search methods or help assess the quality of existing networks with interpretable measures.

How do these two communities’ network planning strategies and suggested network expansions compare when applied to the same network expansion problem? Here, we aim to bridge this research gap by directly comparing the suggested expansion steps from a state-of-the-art mathematical programming model recently published by Paulsen & Rich^7,8^ to a dynamic percolation model suggested by Steinacker *et al.*^32^ for planning the cycling superhighway network of Copenhagen with the same underlying network and routing data. Although the analysis of the results of the mathematical programming model indicates a small optimality gap, its practical implementation remains challenging due to its complexity and reliance on various inputs that are difficult to predict accurately. This raises concerns about its robustness when inputs are uncertain. By contrast, the simpler strategy proposed by^32 ^will have a larger optimality gap. However, it is easier to implement and benefits from a modular structure, enhancing its adaptability to diverse data availability settings. Based on the comparison of these two approaches, this paper presents three main contributions: Firstly, we show that the dynamic percolation model^32^ is extendable to a wide range of objective functions, including an approximation of the social welfare function used in the mathematical programming model^7^. Secondly, if the underlying data is known, the different expansion strategies render broadly similar overall performance with quality differences of less than ${9}\%$. Differences predominantly show as small variations in the specific ordering and the geographical distribution of investments. Thirdly, the more complicated models, here exemplified by the direct optimization model with a complex welfare function, tend to be more susceptible to the uncertainty of input data. This exemplifies the bias-variance trade-off in predictive planning and suggests that more complex models do not necessarily render more reliable predictions in practice.

## Network setting and planning approaches

### Copenhagen cycle superhighway network

**Fig. 2:**
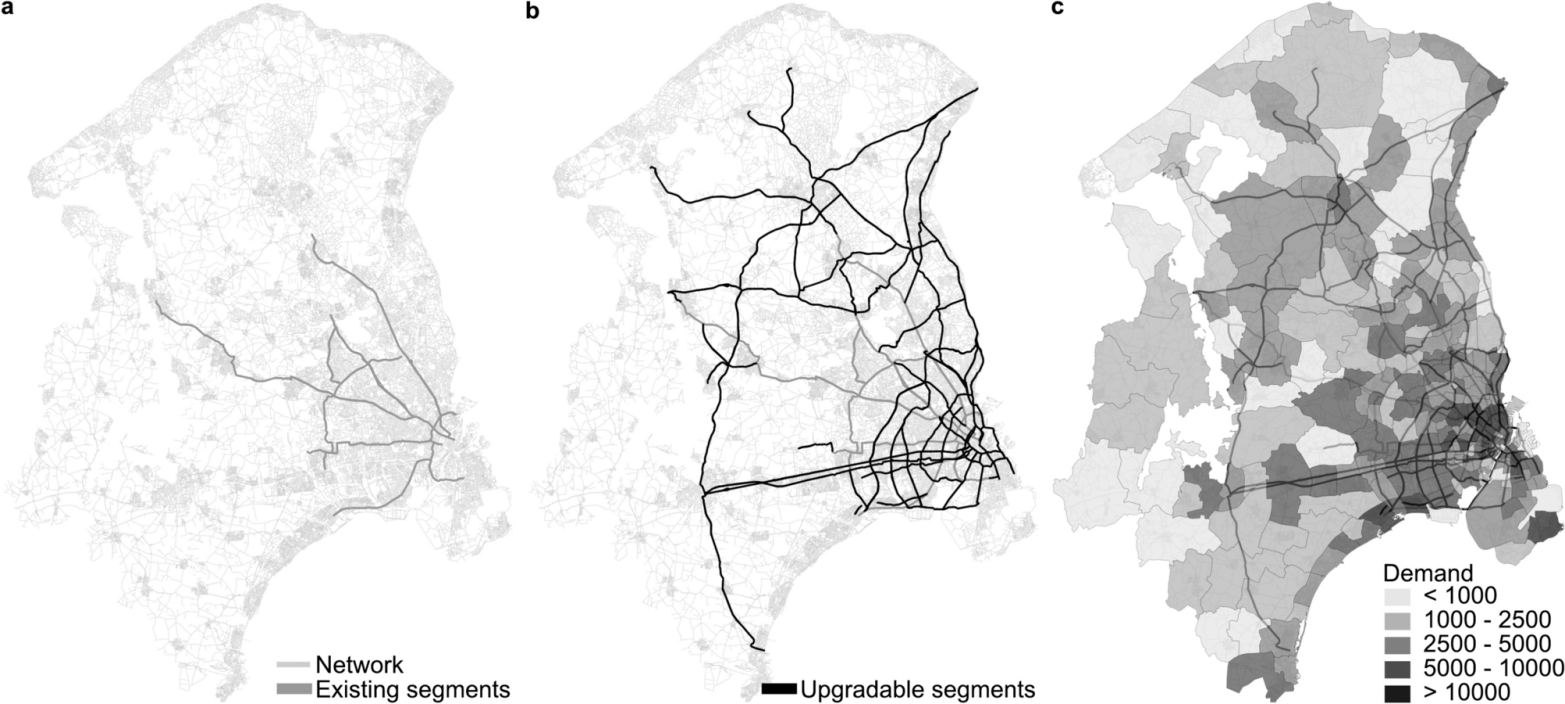
Cycle superhighway network in the greater Copenhagen area. (**a**) Existing infrastructure. Cyclists travel on all links of the network, representing streets and bike paths (light gray) and existing cycle superhighways (dark gray). (**b**) Potential extensions to the cycle superhighway network. Each of the 202 segments of the planned cycle superhighway (black) includes multiple edges of the underlying network. (**c**) Cycling demand. Demand is distributed over 258 origin and destination zones, with the trips starting and ending at the network node closest to the centroid of each zone for a total of $52\,808$ origin-destination-pairs with non-zero demand. The shading denotes the number of cycling trips starting in each zone in the current network state (see Methods for details).

Planning of the Copenhagen cycle superhighway network constitutes a large-scale network planning application. The existing network consists of 460 km of cycle superhighways, mainly in the urban area of Copenhagen (Fig. 2a). Planned extensions of the network include $S = 202$ additional segments with a total length of 1876 km across the greater area of Copenhagen (Fig. 2b). A before-and-after study by Skov-Petersen *et al.*^35^ of Copenhagen’s supercycle highway infrastructure interventions has previously demonstrated the significant benefits of network upgrades and their positive impact on cycling demand, thus highlighting the importance of planning future network extensions.

In our model, each planned segment upgrade encompasses multiple edges that either need to be upgraded from the underlying street network or represent entirely new connections to be added to the network. Cyclists may travel on all links in the street and bike path network, preferentially using faster, more comfortable routes along cycle superhighways and bike paths. Here, we employ a simplified shortest path route choice model with empirical velocities to efficiently enable repeated routing calculations as the network changes during the planning simulations. The demand for cycling is modelled as a zone-based mode choice model with $52\,808$ origin-destination pairs (Fig. 2c) further divided into nine cyclist types separated by fitness and the type of bicycle^36^. See Methods for more details on the model setting and parameters.

We consider the expansion of the cycle superhighway network over a planning horizon of 50 years. Individual segments are added in order of priority, with an annual budget of $B = 7.5$ million EUR for construction and maintenance of the network, carrying over the remaining funds to the next year. In all scenarios, the evaluation period is long enough to allow all segments to be constructed with the total allocated budget.

### Network planning

In a world of perfect information, having more input data can improve models by providing a more detailed representation of objectives and outcomes. However, these added details come at a cost – they increase computation time and demand more effort in data collection and projection. What is often overlooked is that such optimization models are subject to the bias-variance trade-off^37^, a concept from statistics and machine learning. This trade-off suggests that while complex models may excel at replicating known data, they are often poor at making accurate predictions on new, unseen data. The comparison in this paper provides a compelling example of this trade-off, showing that while a highly detailed optimization approach performs well with perfect information, it struggles when input data is uncertain. Below we consider two extremes, one represented by the all-inclusive direct optimization approach and one by a simple bikeability index.

#### Network quality measures

##### Net present value

We evaluate the societal benefits of the cycle superhighway network in terms of its cumulative net present value (NPV) over the 50-year planning horizon. The net present value combines the direct benefits to the individual cyclists represented by travel time benefits (TB), reduced societal costs due to health benefits (HB), the built segments’ construction cost (CC) and maintenance cost (MC), and the remaining scrap value (SV) at the end of the 50-year planning horizon^8^. The individual terms are explained in Tab. 1 and more details are provided in the Methods section. Together, the net present value

1 $$\begin{aligned} \textrm{NPV}&= {\textrm{TB}} + \textrm{HB} - \textrm{CC} - \textrm{MC} + \textrm{SV} \end{aligned}$$

represents the cumulative societal benefits and costs of the network extensions.

**Table 1:** Summary of NPV components and their explanations.

**Term**	**Explanation**
$\textrm{TB}$ (Travel Time Benefits)	Measures the effective travel time savings for cyclists due to the planned expansion compared to the baseline network. Represents the individual economic benefits of improved infrastructure.
$\textrm{HB}$ (Health Benefits)	Measures the positive health effects of increased total distance cycled by the population due to induced demand compared to the baseline network. Represents reduced healthcare costs and overall societal well-being improvements.
$\textrm{CC}$ (Construction Cost)	Measures the financial investment required to build the new cycle superhighway network segments. This is a one-time infrastructure cost.
$\textrm{MC}$ (Maintenance Cost)	Measures the ongoing financial expenditures required to maintain the infrastructure over the 50-year planning horizon.
$\textrm{SV}$ (Scrap Value)	Measures the discounted monetary value of the constructed infrastructure remaining at the end of the planning period. Represents the residual worth of assets after 50 years.

Typically, the travel time savings make up about one-third of the positive contribution to the net present value. Health benefits contribute to the remaining two-thirds. Although estimates of health benefits are subject to considerable uncertainty, primarily due to the challenges of measuring long-term mortality effects, they are incorporated into the official Transport Economic Unit Prices^38^ and must be accounted for in transport appraisal studies. The negative contributions of construction and maintenance costs for bicycle infrastructure are comparatively small, amounting to less than ${10}\%$ of the absolute net present value. However, due to a limited budget, construction and maintenance costs strongly affect which and how many cycle superhighway segments are added to the network each year. For Eq. 1 to be exact, the inputs need to be known precisely and deterministically for the entire investment horizon. If, on the other hand, inputs are considered uncertain, these errors will propagate over the years and render much less robust solutions which could perform worse compared to simpler variants.

##### Bikeability

The bikeability index^32^ is an example of a simpler network quality measure representing a non-monetary valuation based on improving cycling travel time and demand in the network. While it does not represent the monetary investment or full societal benefit of the network, it serves as a simple comparison of the efficiency of a bike path network in enabling direct travel and forms the basis of the network percolation approach introduced in Steinacker *et al.*^32^. To define the bikeability, we first define a loss function $L \ge 0$ for a single trip $\omega$ with travel time $\tau$ in the current network *G* as the integral under the demand curve $n(\omega , \tau )$,

2 $$\begin{aligned} L(\omega , G)&= \int _{0}^{\tau } n(\omega , \tau ^\prime ) \, \textrm{d} \tau ^\prime \,, \end{aligned}$$

The loss function decreases when new bike paths decrease the travel time $\tau$ of the trip, with a stronger effect the larger the number $n(\omega , \tau )$ of cyclists on the trip. By summing up the loss functions $L(\omega ,G)$ for all individual trips $\omega$, we obtain a network-wide loss function

3 $$\begin{aligned} L(G) = \sum _{\omega } L(\omega , G) \,. \end{aligned}$$

To evaluate the progress from the base network $G_\textrm{base}$ to the fully upgraded network $G_\textrm{full}$, we define the bikeability *B* of an intermediate cycle superhighway network *G* as the normalized improvement of the network-wide loss function *L*,

4 $$\begin{aligned} B(G)&= \frac{L\left( G_\textrm{base}\right) - L\left( G\right) }{L\left( G_\textrm{base}\right) - L\left( G_\textrm{full}\right) } \,, \end{aligned}$$

such that $B(G_\textrm{base}) = 0$ for the base network and $B(G_\textrm{full}) = 1$for the fully upgraded network^32^ (see also Supplementary Note 1, including Fig. S1).

#### Network optimization strategies

##### Direct optimization

Direct optimization is, by nature, impractical for large-scale problems with extensive geographical coverage and long planning periods. As a result, applied solution methods are often either based on probabilistic sampling, e.g. simulated annealing^39^, genetic algorithms^22^, or simplify the optimization problem into set-covering problems^20,21^. As a reference approach in this paper, we employ iterative batched optimization of newly constructed cycle superhighway segments for each year of the planning period to maximize the net present value at the end of the planning period^8^. The method solves a mathematical program in each time period to determine the most societally profitable segments. If no such segments exist, no segments are constructed.

Additionally, we test a simplified greedy optimization approach based on a linear interpolation of cyclists’ route choices between the original and the fully upgraded network. This greedy approach iteratively constructs the entire network by sequentially adding the most valuable cycle superhighway segment regarding its approximated net present value contribution per construction cost. The linearized evaluation of the objective function avoids the explicit calculation of the routes during the optimization, thus enabling a faster solution to the optimization problem. However, evaluating the quality of the resulting networks still requires the calculation of the routes of all cyclists in each network state.

Expanding the network from the base state, both optimization algorithms explicitly include time-dependent information in the evaluation, such as budgets and discounting future benefits to extrapolate the value of the objective function at the end of the planning period. The methodology underlying both optimization approaches is described further in the Methods section below.

##### Dynamic backward percolation

Percolation describes the emergence or breakdown of macroscopic connectivity in networks as links are added or removed. Dynamic percolation methods have initially been suggested to reveal the community structure of a network by iteratively removing links with the highest betweenness to disconnect the network as efficiently as possible^40^. Similar approaches have since been applied to understand the structure of aviation networks by iteratively removing unprofitable connections^41^.

**Fig. 3:**
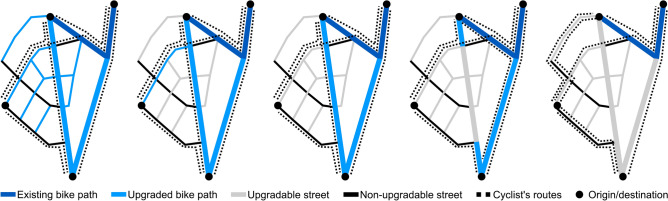
Dynamic backward percolation^32^. Thick lines represent main streets, thin lines represent residential streets. The color denotes the type of bike infrastructure: (gray and black) streets without a bike path, (dark and light blue) existing and newly constructed bike paths, respectively. Starting from a network where every upgradable street (gray) is equipped with a bike path (light blue), we first remove all unused bike paths given the demand distribution and the routes of all cyclists (dotted lines). We then iteratively remove the least important newly constructed bike path and recompute the routes of all affected cyclists. The process ends when all newly constructed bike paths have been removed and only the existing infrastructure (dark blue) remains. Reversing the order of bike path removal provides a prioritization for the bike path network extensions.

Recently, a bike path planning algorithm based on backward percolation was presented^32^. Starting from a fully upgraded network, the algorithm iteratively removes the least important bike path, generating a sequence of extensions in inverse order (Fig. 3). This inverse approach avoids reinforcing inefficient route choices of cyclists in an incomplete network (e.g., taking detours to avoid busy streets without a bike path). In contrast, static or forward percolation approaches often lock cyclists in suboptimal routes when selecting network extensions based only on the route choices in the sparse base network of bike paths^30,33^. This is especially detrimental when planning network extensions with insufficient infrastructure.

The dynamic nature of the approach means that the routes of all cyclists are recomputed after a bike path has been removed. The algorithm thus automatically tracks the routes and demand as a function of the current bike path network, enabling online evaluation of the network quality. In addition, this makes the approach highly flexible and allows for the computation of almost arbitrary importance measures and route choice models. However, dense demand or complex models may result in long computation times. Additionally, due to the inverse construction, the order of extensions is only fixed once the algorithm has finished. Since this order is crucial to determine the cumulative maintenance cost and required budget, it is impossible to explicitly compute time-dependent evaluation functions like the net present value during the planning simulation.

In this paper, we test this backward percolation approach with three different importance measures for the segments that represent successively more accurate approximations of their contribution to the net present value of the network. The first measure $Q_\textrm{pen}(s)$ quantifies the weighted travel time penalty for cyclists when removing a segment *s* relative to the length of the segment. The second measure $Q_\textrm{stat}$ is derived from an explicit linearization of the change in travel time benefits relative to the construction costs of the segment, again assuming static routes and demand. Finally, assuming only static routes but allowing dynamic demand, the third measure $Q_\textrm{dyn}$ is derived from a linear approximation of travel time benefits and health benefits, capturing the majority of the change in net present value. All measures are explained in more detail in the Methods, derivations are provided in Supplementary Note 2 (including Fig. S2) and Supplementary Note 3, respectively.

## Results

In the following analysis, we evaluate the performance of the different network planning approaches by focusing on four aspects: First, we assess the overall societal network performance. Next, we examine the geographical distribution of network expansion. Third, we analyze the timing of individual investments. Finally, we investigate the robustness of the methods with respect to uncertain input data.

### The overall welfare performance

**Fig. 4:**
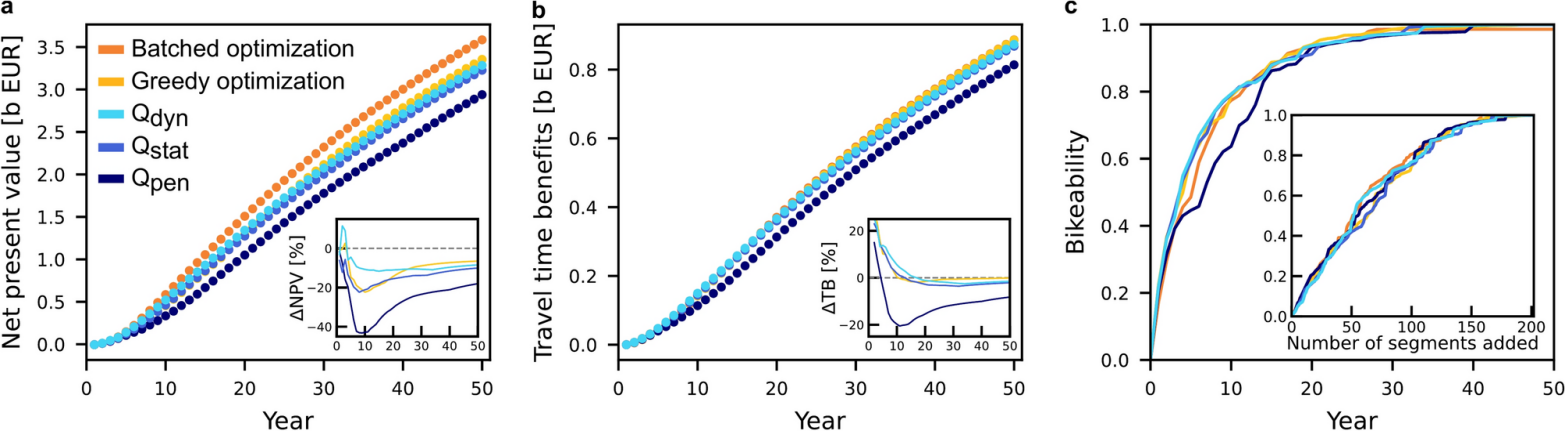
Similar performance of planned networks from dynamic backward percolation and direct optimization. (**a**) Net present value (NPV), measuring the cumulative welfare benefits, including travel time savings, additional cycling demand, and health benefits as well as construction and maintenance cost of the bike paths [Eq. (1)]. (**b**) Travel time benefits (TB), measuring the cumulative time saved by all cyclists [see Methods, Eq. (8)]. Insets in panels (a) and (b) show the difference in NPV and TB relative to the batched optimization approach. (c) Bikeability *B*, measuring the relative improvement of travel time and demand between the current and fully built state [Eq. (4)]. (inset) Bikeability *B* as a function of the number of segments added. The different approaches achieve similar network performance (panel a-c). Both direct optimization approaches achieve a higher net present value at the end of the 50-year planning horizon as their explicit optimization goal. The networks suggested by the backward percolation approach with the dynamic-demand evaluation function $Q_\textrm{dyn}$ provide a slightly larger net present value and welfare gain during the first years of the planning period compared to the batched direct optimization (insets). All approaches achieve a similar bikeability as a function of the number of added segments, suggesting a similar ordering of segment prioritization (inset in panel c). However, the simple evaluation measure $Q_\textrm{pen}$ for the backward percolation approach falls behind in all measures when evaluated as a function of time, highlighting the importance of maintenance cost and budget constraints.

We compare the performance of all network expansion strategies in terms of the net present value of the welfare function and the bikeability index of the networks (Fig. 4). Overall, dynamic backward percolation and direct optimization achieve very similar results across the whole planning period.

As expected, since it more explicitly optimizes for it, direct optimization achieves a higher net present value at the end of the 50-year planning horizon (Fig. 4a). The backward percolation with a dynamic-demand evaluation function $Q_\textrm{dyn}$ initially achieves higher net present value compared to batched optimization in the first 3 years of the planning period (up to 12% in year 2, compare Tab. 2). The initial gains in the early years of the planning period (Fig. 4b), however, are quickly offset by the higher maintenance costs of the constructed segments. By the end of the planning period, the net present value achieved with the backward percolation approach trails behind by about 8 to 9% compared to batched optimization. Greedy optimization performs significantly worse for the first 25 years (up to 22% in year 10). Still, it closes that gap to about 6 to 7% by the end, achieving a slightly higher net present value than backward percolation. With increasing simplification of the segment evaluation, the performance of the backward percolation approach decreases. While the static-demand approximation $Q_\textrm{stat}$ still achieves comparable results to $Q_\textrm{dyn}$, the simplified evaluation function $Q_\textrm{pen}$ falls behind by up to 20% after 50 years.

The network performance with respect to the welfare function and the bikeability (Fig. 4b,c) behaves similarly across all approaches, with differences between direct optimization and percolation approaches becoming smaller for simpler network quality measures. Interestingly, as a function of the number of segments added, the bikeability index is almost identical across all methods. Here, even the simplest evaluation function $Q_\textrm{pen}$ performs as well or slightly better than the other measures (Fig. 4c inset), but loses this advantage when the evaluating the bikeability as a function of time (Fig. 4c), emphasizing the importance of including construction and maintenance costs for budget-constrained network expansions.

**Table 2:** Network costs and performance. Comparison of key observables of the planned networks by batched direct optimization (DO) and by backward percolation (BP) with the dynamic-demand evaluation function $Q_\textrm{dyn}$. After year 3, both planned networks have similar total length and maintenance costs (MC). Though the construction costs (CC) of BP is 13% smaller compared to DO, it achieves 9% larger net present value (NPV) due to larger travel time benefits (TB). After year 10, both networks still have a similar total length with almost identical construction costs. Yet, the network planned by BP has 5% higher maintenance costs, reducing the available budget for future investments. Also, DO already achieves a 11% higher net present value over the BP despite lower welfare gains due to its advantage in health benefits (HB). After year 30, the batched direct optimization no longer adds additional segments to the network and maintains a net present value approximately 8 to 10% higher than the backward percolation approach. See Supplementary Note 4, Fig. S3 for the cost and performance of all five approaches.

	Year 3		Year 10		Year 30		Year 50		$G_\textrm{full}$
	DO	BP	DO	BP	DO	BP	DO	BP	
Total length built [km]	338	350	693	705	1331	1388	1331	1876	1876
Relative length built [percent]	18.01	18.65	36.94	37.58	70.92	73.98	70.92	100.00	100.00
$\textrm{CC}_\textrm{rel}$ [percent]	6.86	6.01	20.58	20.56	54.34	57.41	54.34	100.00	100.00
$\textrm{NPV}$ [m EUR]	41.56	45.35	584.75	522.06	2321.80	2074.91	3585.40	3284.11	-
$\textrm{TB}$ [m EUR]	14.66	17.82	141.64	149.49	575.12	562.41	886.72	873.28	-
$\textrm{HB}$ [m EUR]	43.00	41.87	498.14	427.20	1912.15	1679.53	2929.26	2683.81	-
$\textrm{CC}$ [m EUR]	15.18	13.28	45.50	45.46	120.14	126.92	120.14	221.10	221.10
$\textrm{MC}$ [m EUR]	0.72	0.73	3.08	3.24	9.30	9.78	9.30	18.20	18.20

### Geographical distribution

**Fig. 5:**
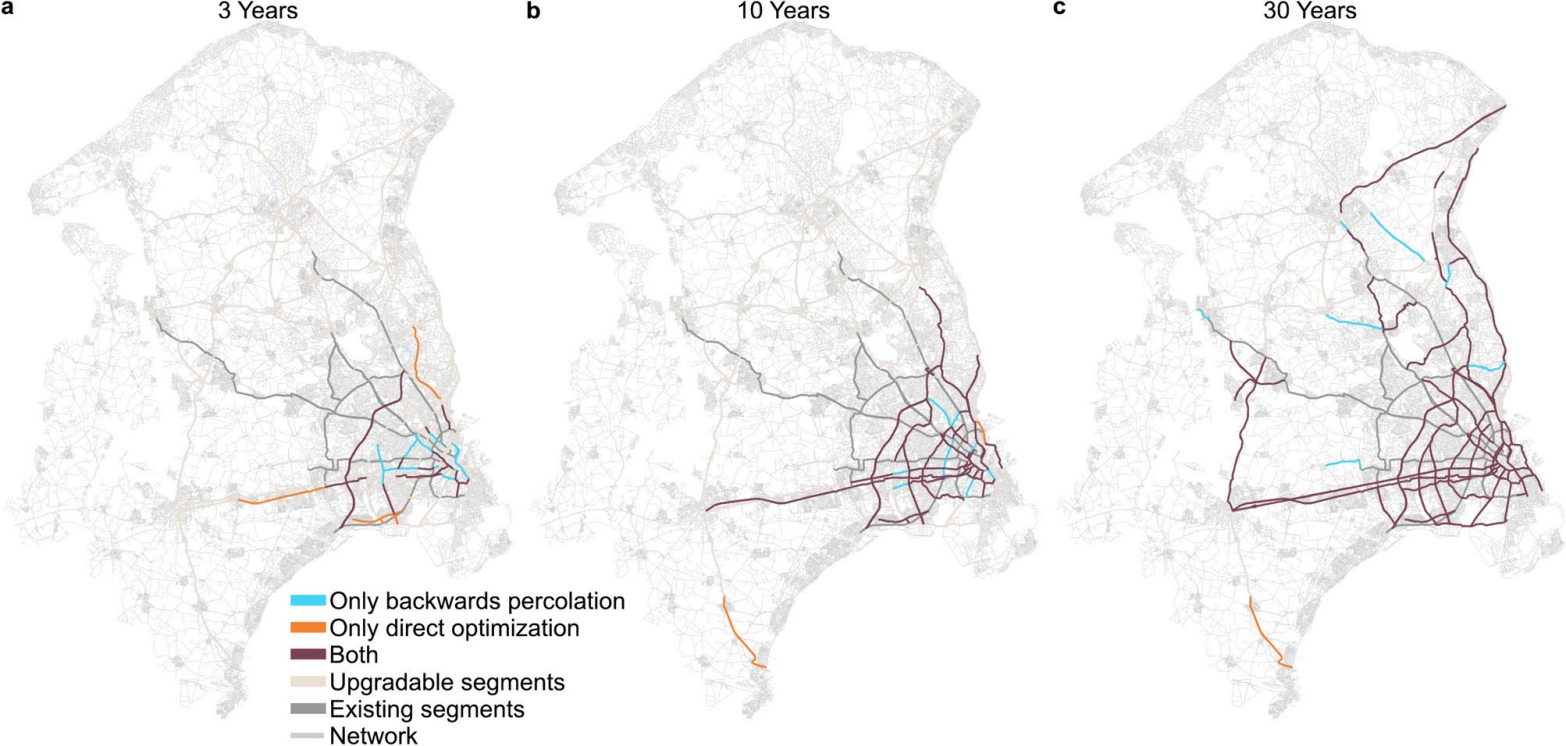
Similar planned networks from dynamic backward percolation and direct optimization. (a-c) Comparison of the planned networks by direct optimization and by backward percolation with the dynamic-demand evaluation function $Q_\textrm{dyn}$ after (**a**) 3 years, (**b**) 10 years, and (**c**) 30 years (compare Fig. 2a,b, see Tab. 2 for a quantitative comparison of the costs and benefits of the networks). The planned networks largely coincide (dark red), especially in the urban area of Copenhagen. The direct optimization approach constructs a segment in the south of Copenhagen that remains disconnected from the rest of the already built or existing cycle superhighway network but provides a large net present value benefit (colored or gray, panels b and c).

In the following, we compare the geographical distribution of the generated networks between the best-performing direct optimization and backward percolation approaches. Fig. 5 illustrates the geographical distribution of the proposed expansion after 3, 10 and 30 years (see videos of the network expansion in the Supplementary Material for separate illustrations of the networks of the individual approaches over the 50-year planning period). Initially, in the first three years, both approaches mainly construct relatively short segments in the inner parts of Copenhagen, where demand is highest, and complete a secondary ring around the city (compare Fig. 5a). These segments achieve the largest improvement in net present value with relatively low costs. However, batched optimization already constructs some additional radial segments outside the main city area, whereas the backward percolation approach remains focused on the city center. After 10 years, both approaches have built roughly half of the possible segments. The similarity of the suggested networks is high (Fig. 5b). Intriguingly, the direct optimization approach constructs a single disconnected segment south of Copenhagen. In contrast, the network suggested by the backward percolation approach is slightly denser in the city center, containing more costly segments. While these segments greatly improve the network, their high maintenance cost restricts the options for the backward percolation approach in the subsequent years, especially in these intermediate planning stages (see Tab. 2). This observation explains the better performance of the backward percolation approach in the early years and the loss of performance in the later parts of the planning period because the net available budget is smaller and fewer segments are added annually.

After 30 years, both approaches have constructed nearly all planned segments in the urban area of Copenhagen. The planned networks differ by only eight segments. The direct optimization has already reached its final expansion stage. Some newly built segments in the outskirts remain disconnected from the rest of the cycle superhighway network as the direct optimization approach does not add all possible segments (Fig. 5c, e.g. the segment in the south, which was built very early on in year 5 and some small segments to the north of Copenhagen).

### The timing of investments

**Fig. 6:**
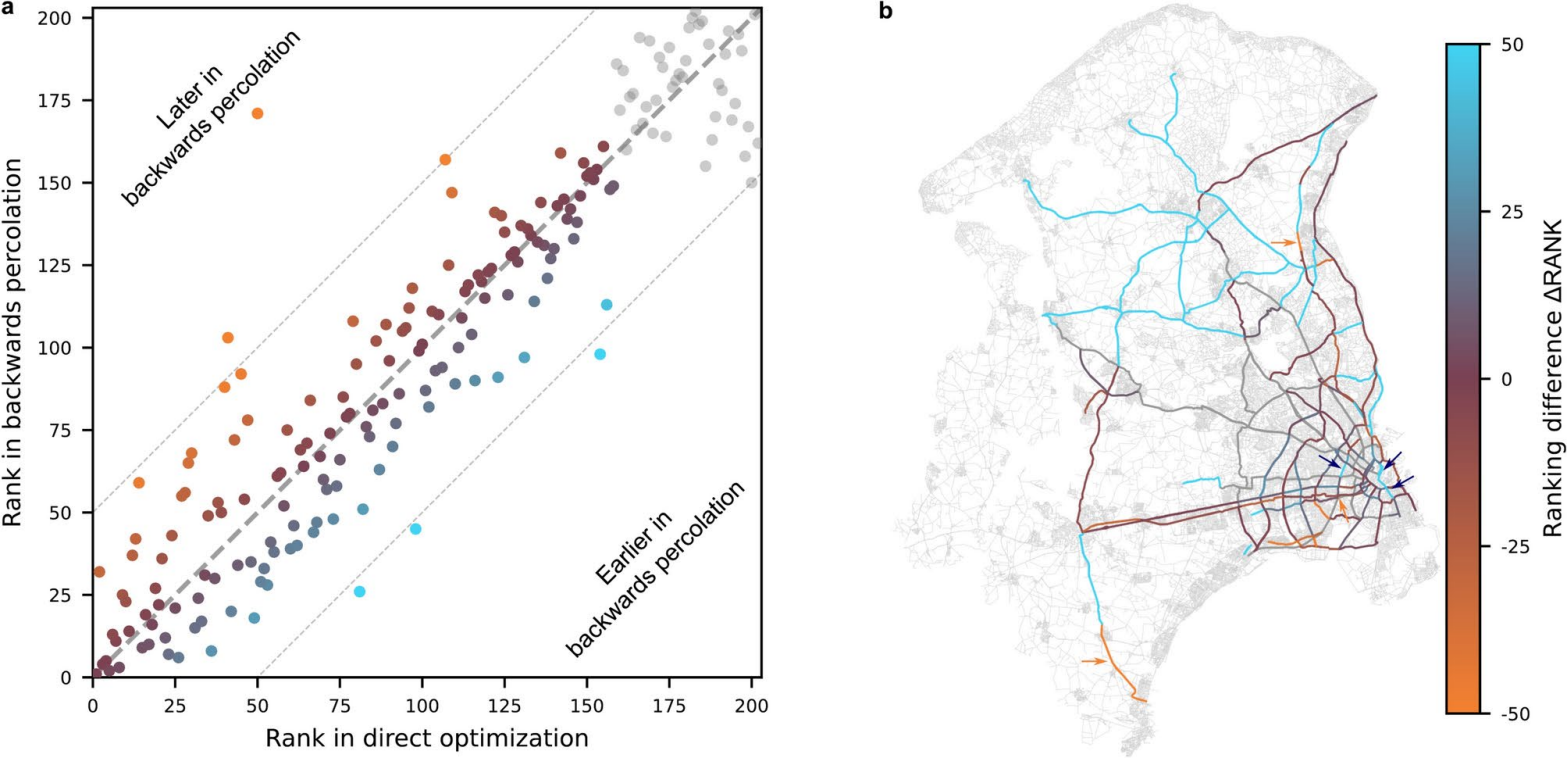
Similar prioritization of cycle superhighway segments in backward percolation and direct optimization. (**a**) Comparison of the priority rank of each link in the suggested build order in the backward percolation and direct optimization approach. Points on the identity (gray dashed line) indicate identical ranking of segments in the build order, points above (below) the line indicate links that are built later (earlier) in the backward percolation approach. Gray points in the top right represent segments not built by direct optimization. The block structure of the figure suggests that both approaches identify a similar set of least important segments. (**b**) Map illustrating the difference in ranking for each segment (blue: earlier in backward percolation; orange: later in backward percolation, compare panel a). Arrows highlight the seven outliers with a ranking difference $|\Delta \textrm{STEP}|\geq 50$ (outside the dashed light gray lines in panel a). Four of the six outliers are located in the urban area of Copenhagen.

While the planned networks are very similar after 30 years, the timing of the investments up to this point differs. To assess these timing differences between the two planning approaches in more detail, we compare the exact ordering of the segment prioritization (Fig. 6). We evaluate the difference in the ranking of the segments by the two approaches,

5 $$\begin{aligned} \Delta \textrm{RANK}(s)&= \textrm{RANK}_\textrm{DO}(s) - \textrm{RANK}_\textrm{BP}(s) \,, \end{aligned}$$

where a smaller rank indicates a higher priority (earlier construction). If the difference $\Delta \textrm{RANK}(s)$ is positive (negative), the segment *s* is considered more (less) important and built earlier (later) in the backward percolation (BP) approach compared to direct optimization (DO). To be able to compute an explicit ranking of all segments for batched direct optimization, we order the segments constructed in the same year by their rank in the greedy optimization and append segments not built to the end of the ranking.

The results confirm the similarity of the networks generated by both approaches. Over 50% of the segments are ranked within a difference $|\Delta \textrm{RANK}|\le 9$ and are built within 2 years of each other. Over 75% are ranked within $|\Delta \textrm{RANK}|\le 20$ and are built within 4 years. There are only six outliers ($|\Delta \textrm{RANK}|\geq 50$, see Fig. 6a), constructed more than 5 years apart by the different planning approaches, one even 29 years. The two outliers built early by direct optimization are located in the perimeter of the planning region (Fig. 6b), notably including the segment in the south that is constructed early and remains disconnected (compare Fig. 5b).

### Robustness of suggested network extensions

**Fig. 7:**
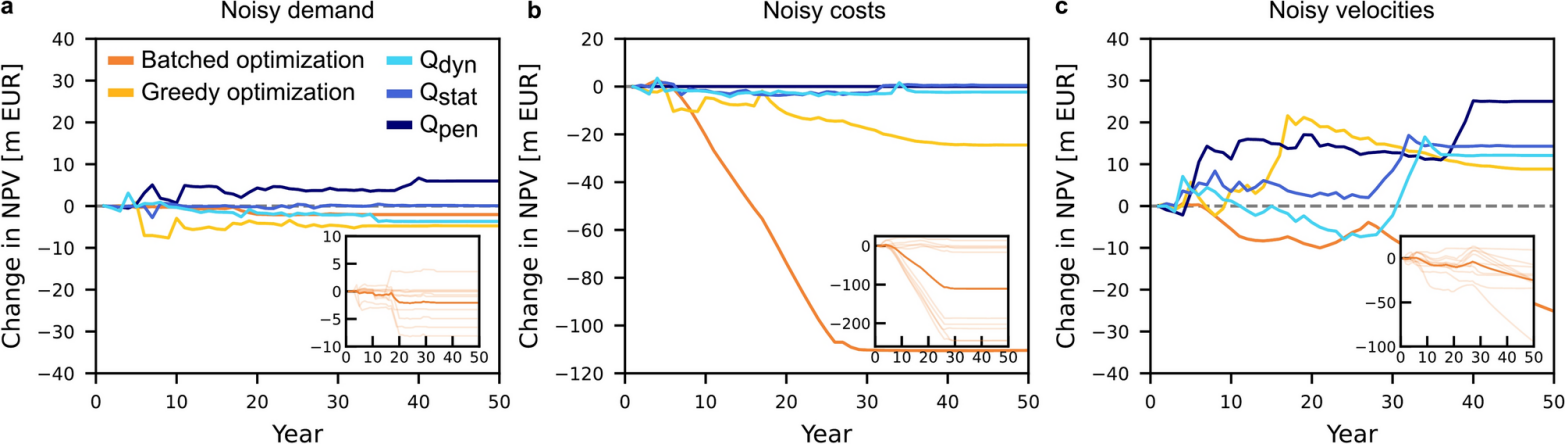
Robustness of suggested cycle superhighway extensions decreases with model complexity. (**a**-**c**) Change of the net present value of the networks planned with noisy data compared to networks planned with the exact parameters for (**a**) noisy demand, (**b**) noisy costs, and (**c**) noisy velocities. The insets show the result for the single runs and the mean, exemplary for the batched optimization. While the simplest importance measure $Q_\textrm{pen}$ of backward percolation results in the smallest net present value (compare Fig. 4a), it is overall the most robust to variations of the input data. With increasing model complexity, the change in net present value increases on average, indicating an effect similar to overfitting of the optimized network expansion order to the input data.

Any network planning application is subject to uncertainty, whether due to changing conditions that cannot be captured in the model or inaccurate and noisy data underlying the planning decisions. Here, we test the robustness of the cycle superhighway extensions with respect to noise in three different parts of the input data by varying the original values by up to $\pm {20}\%$: (i) We consider noisy demand by adding multiplicative noise with a uniformly random factor $\lambda \in [-0.2,0.2]$ to the demand from each zone (compare Fig. 2), adjusting the trip demand for an origin-destination-pair (*o*, *d*) by the average of the noise factor of both the origin and destination zone. (ii) We consider noisy costs by adding multiplicative noise with a uniformly random factor $\lambda \in [-0.2,0.2]$ to each cycle superhighway segment’s construction and maintenance cost independently. (iii) We consider noisy velocity values for the cyclists by adding multiplicative noise with a uniformly random factor $\lambda \in [-0.2,0.2]$ to each speed-cyclist-infrastructure velocity, ensuring that the relative ordering of the speeds inside categories remains the same (compare Methods, Tab. 3). The aim is to test the robustness of the cycle superhighway extension with respect to current uncertain knowledge and misestimation of the model parameters. We compute network extensions for ten samples of noisy input data for each of the three variations and compare the resulting average deviations of the net present value of welfare (Fig. 7), travel time benefits, and bikeability (Supplementary Note 5, Fig. S4). Results of all individual realizations are presented in Supplementary Note 5 Figs. S5 to S10.

To evaluate the robustness of the different methods, we compare the performance of the planned cycle superhighway extensions with noisy input data to the original planning with non-noisy parameters of the respective method (Fig. 7). Inaccurate demand data has almost no effect on the resulting network quality for all five methods. This is likely due to the large number of origin-destination pairs averaging out the impact of the noise.

Uncertain segment costs have a more significant influence on the planned network performance, especially for the direct optimization approaches. In contrast, the backward percolation with the simplest quality evaluation function $Q_\textrm{pen}$ is not affected at all, since it does not explicitly consider segment costs. The batched optimization is highly affected and loses up to 240 m EUR corresponding to 2/3 of its additional net present value compared to the backward percolation models (Fig. 7b inset).

Noisy velocities have a strong impact across all approaches, likely because the velocities play a key role in routing the cyclists and especially since a binary shortest path route choice model is used. Here, even planning based on the simplest importance measure $Q_\textrm{pen}$ is strongly influenced, as it heavily relies on the ratio of the velocities to define the importance of a cycle superhighway segment. Intriguingly, the quality of the planned networks, on average, improves slightly for all approaches except batched optimization, which already achieves a higher net present value than the other approaches.

For a detailed explanation of the robustness dynamics, we have to differentiate between two areas: (i) general, approach-based differences/dynamics and (ii) specific, data- and network-dependent differences.

In general, the lower robustness of the batched approach can be attributed to its near-optimal performance with the base non-noisy input data. Any change in the proposed ordering is more likely to (strongly) reduce the benefits. In contrast, for the simpler models, including the greedy optimization, changes in the ordering are more likely to also result in better performance. At the same time, the network evaluation measures used in these simpler models also depend on fewer parameters and combine fewer terms, resulting in overall smaller fluctuations.

For this specific network, a large change in the net present value is caused by a single (long) segment of the cycle superhighway (visible, for example in the qualitative change of the trajectories in the inset in Fig. 7b). Delaying the construction of this segment causes a significant decrease in the net present value of the resulting network. However, due to the size and cost of the segment, estimates of the benefits and its impact on routing decisions are unstable. Consequently, the evaluation of the segment changes drastically and it is often built at different times, especially with noisy costs or velocities.

Overall, variations in the build order and correlation of the ranking of the segment importance (see Supplementary Note 5, Fig. S5) confirm the qualitative observations: more complex optimization algorithms are typically more susceptible to inaccurate input data. These results highlight the benefits of simplified, abstract models when data is known to be uncertain.

## Discussion

Planning efficient network extensions is a highly complex problem across all applications due to multiple, interacting, and often opposing constraints and requirements^39,42^. Here, we have directly compared two complementary network planning approaches for the design of cycle superhighway extensions with the same underlying network and demand data. While direct optimization approaches achieve a higher net present value, our results show that abstract conceptual planning approaches may achieve comparable performance with simpler and often more robust evaluation functions.

Both approaches have advantages and disadvantages, but complement each other in their applicability for different use cases. The backward percolation approach produces more reliable solutions to the problem in case of uncertain and absent input data. This makes it useful as a first step in the network planning phase. However, the backward planning makes it impossible to evaluate properties depending explicitly on the construction time of individual infrastructure elements, such as the total maintenance costs until the end of the planning period. In contrast, the direct optimization approaches construct the network from the ground up with explicit reference to the budgetary constraints^7,8^. This method achieves better performance for the explicit optimization goal and is likely more suitable for constructing accurate expansion plans later in the planning process when all parameters and conditions are known sufficiently accurately. However, simplifications to the optimization problem, like the linearization of travel time improvements (see Methods), seem to result in more disconnected bike paths in intermediate network stages compared to other approaches that focus on conceptual measures and put more emphasis structural properties of the networks. While we have only tested the robustness of the suggested network extensions to variations in the input data, planning problems often suffer from increasing uncertainty of external conditions and model parameters during the planning period. A valuable extension of this research would be exploring how such progressively increasing uncertainty affects solution strategies, especially given the time horizons considered. Additionally, a route choice model that does not solely rely on travel speeds would be a beneficial extension. Yet, such a route choice model still needs to be computationally efficient when used with a rapidly changing network, excluding standard logit models that rely on predefined choice sets.

Neither approach fully automates the planning process, as these processes often exhibit multiple stages and revisions. Furthermore, the prioritization and actual implementation of extension measures requires much more information than can be represented in any algorithmic model. Not only is the participation of residents and businesses necessary in the planning process^43,44^, but as other modes of transport are often impacted by the construction of new bike paths, a comprehensive urban planning perspective has to include a multi-modal evaluation^24^. Additional robustness analyses may provide more detailed insights on the variability of the planned extensions with respect to such model extensions, for example more detailed route choice models or additional optimization criteria representing costs and benefits for other road users and stakeholders.

Regardless of the potential extensions, the suggested networks may serve as a basis for planning and supporting an efficient prioritization of planned network extensions in such a multi-stage process. After the identification of possible extensions, which are politically and technically feasible, our model could help to rank and prioritize these planned extensions. With this ranking, city planners could start detailed planning, including community feedback and add-on effects on other road users. Our algorithmic planning approaches may also help to identify critical extensions that, if proven unfeasible or too costly in these stages, would result in large changes of the suggested network expansion. This might help to focus efforts on important segments that require detailed evaluation early in the planning process to identify robust long-term expansion strategies.

In general, both network planning approaches may (with some adaptions) also be applied to general infrastructure networks^45^, including multi-modal transport infrastructure^46–48^, charging infrastructure for electric vehicles and power grids^49,50^, or planning resilient infrastructure for climate change^51–53^. While a wide range of other methodologies, such as reinforcement learning often used in approximate dynamic programming, have been applied to solve network planning problems in these different contexts, the scalability of many methods to large network contexts emerging in many of these applications remains an open question.

Overall, our results show that conceptual network design approaches from network science may compete with direct optimization methods and complement existing approaches. Together, both approaches may help design more efficient bike path networks to support cycling and the transition to more sustainable urban mobility.

## Methods

### Copenhagen cycle superhighway model

**Network** For the numerical implementation, we represent the street, bike path, and cycle superhighway network as a directed graph $G = (V,E)$. The vertices represent intersections as well as intermediate points along single streets, bike paths, or cycle superhighways. The edges *E* represent the directed connections along the streets, bike paths, or cycle superhighways. Each directed edge $e \in E$ is assigned a physical length $l_e$ and one of three infrastructure categories (street, bike path, cycle superhighway), determining the travel speed of cyclists on the edge (see below).

We distinguish between the base network $G_\textrm{base} = (V_\textrm{base}, E_\textrm{base})$ without any of the proposed cycle superhighway upgrades (Fig. 2a) and the planned upgrades $G_\textrm{build}$ (Fig. 2b). Their combination constitutes the fully upgraded network, $G_\textrm{full} = G_\textrm{base} \cup G_\textrm{build}$. Over the course of the optimization, we add a total of $S = 202$ cycle superhighway segments $E_s$, $s \in \{1,2,\ldots ,S\}$, each consisting of multiple individual edges, $E_s = \{e_1(s), e_2(s), \ldots \}$. These edges may represent upgrades to existing edges in the network $G_\textrm{base}$ or entirely new connections. When a cycle superhighway segment is added to the graph *G*, we add its edges and vertices to the network (if the edges did not exist in the network yet) or update the infrastructure types of the edges included in the segment to cycle superhighway (if the edges were already part of the network as streets or normal bike path).

The full network $G_\textrm{full}$ (compare Fig. 2a,b) has $|V|= 191\,448$ vertices and $|E|= 453\,433$ directed edges with a total length of approximately 33678 km, double-counting directed edges in both directions along the same path. A large part of the network is already covered by the normal bike path network, accounting for 9894 km (29.4%) of the length of the network. The existing cycle superhighway network makes up 460 km (1.4%) with the proposed upgrades adding 1876 km (5.6%). Of the proposed segments, 1666 km are upgrades based on existing links within the base network and 210 km are completely new infrastructure.

The construction cost^54^of the proposed cycle superhighway segments range between 5468.73 €/km and 1245096.76 €/km, with an average of 138978.02 €/km (median 96972.49 €/km). Their annual maintenance cost^54^ range between 636.41 €/km and 98621.53 €/km, with an average of 11484.49 €/km (median 7441.89 €/km).

**Demand** The demand model consists of $52\,808$ origin-destination pairs between 258 starting and end points (compare Fig. 2c), representing the annual number of cyclists^36^. The demand for each origin-destination pair is further split into 9 different cyclist types, reflecting the fitness or general speed of the cyclists (slow, medium, fast) and the type of bicycle (regular bicycle, e-bike and speed pedelec)^36^. Normal bikes make up 95% of the trips, e-bikes 4.5%, and speed pedelecs account for the remaining 0.5%. We assume that 50% of cyclists belong to the medium speed type and 25% to the slow and fast type, respectively. Each cyclist type is assigned a velocity *v* for each of the three infrastructure categories of edges in the network (street, bike path, cycle superhighway), see Tab. 3, summarizing the actual travel time as well as perceived comfort and safety of travel along the edge.

We model induced demand as a function of the travel time $\tau (\omega ,G)$ of cyclists for a trip-cyclist-type combination $\omega$ in the network $G$ with a logit model^55^. The travel demand $n(\omega , G)$ changes as

6 $$\begin{aligned} n(\omega ,G) = n(\omega ) \, P(\omega , G) = n(\omega ) \, \frac{e^{-\beta \,\tau (\omega ,G)}}{e^{-\beta \,\tau (\omega ,G)} + e^{-\beta \,\tau _\textrm{other}(\omega )}} \end{aligned}$$

where $n(\omega )$ denotes the total travel demand for the trip $\omega$ and $\tau _\textrm{other}(\omega )$ the constant travel time for the trip $\omega$ with other modes of transport. The parameter $\beta \approx {-0.0518}\,\textrm{h}^{-1}$ is the parameter that describes the demand sensitivity. This parameter is calibrated to render elasticities similar to the ones used by Hallberg *et al.*^36^and Paulsen & Rich^8^.

For the evaluation of the net present value, we include population growth. The trip demand in a network *G* at time *t* is given by

7 $$\begin{aligned} n(\omega ,G,t) = \gamma (t) \, n(\omega ,G) = \gamma (t) \, \frac{n(\omega ,G_\textrm{base})}{P(\omega ,G_\textrm{base})}\, \frac{e^{-\beta \,\tau (\omega ,G)}}{e^{-\beta \,\tau (\omega ,G)} + e^{-\beta \,\tau _\textrm{other}(\omega )}} \end{aligned}$$

with the prefactor $\gamma (t)$ capturing overall population growth, with $\gamma (0) = 1$ and increasing by approximately ${0.2}\%$ per year for a total increase of ${7}\%$ by the end of the 50-year planning period^56^. Here we write the total demand $n(\omega ) = \frac{n(\omega ,G_\textrm{base})}{P(\omega ,G_\textrm{base})}$ as a function of the demand $n(\omega ,G_\textrm{base})$ and the fraction $P(\omega ,G_\textrm{base})$ of cyclists in the base network.

**Route choice model **Modelling cyclist route choices in a changing network environment is in itself a complex problem^57^. Cyclist routing is largely unaffected by congestion, as low space requirements and cyclist volumes keep travel times stable, with uncertainties mainly from intersection delays^58^. We, therefore, consider each trip individually and adopt a simplified shortest-path choice model. This has the added benefit of avoiding complications and bias introduced by the explicit generation of choice sets as the network changes over time. Hence, cyclists travel through the network following the shortest path with respect to their travel time, determined by their bike type and speed on the different types of network edges as explained above (Tab. 3, derived from empirical speed measurement from cyclists in Denmark on different infrastructure types^36^). In addition to the travel time along the network edges, we add time penalties for complex intersections. These penalties are the same for all cyclist types, adding 30 s for large traffic light-controlled intersections, 5 s for roundabouts, and 0 s for the remaining ones (including, for example, intermediate vertices along paths, intersections between small residential streets, and on- and off-ramps of the cycle superhighway network)^36^.

**Table 3:** Travel speeds in km/h of cyclist types based on Hallberg *et al.*^36^.

Bicycle Type	Speed Type	Infrastructure Type		
		Street	Bike Path	Cycle Superhighway
Regular Bicycle	Slow	13.6	15.1	16.6
	Medium	16.3	17.8	19.3
	Fast	19.1	20.8	22.5
E-bike	Slow	15.6	17.1	18.6
	Medium	18.3	19.8	21.3
	Fast	21.1	22.8	24.5
Speed Pedelec	Slow	22.6	24.1	25.6
	Medium	25.3	26.8	28.3
	Fast	27.3	29.8	31.5

### Net present value of the cycle superhighway network

We measure the societal benefits of the cycle superhighway network extensions in terms of the net present value [NPV, Eq. (1)] of the network *G*^7,8^. The net present value includes five components:

(i) The annual travel time benefits (TB),

8 $$\begin{aligned} {\textrm{TB}}(G,t) = \sum _\omega \zeta (\omega )\,\frac{n(\omega ,G_\textrm{base}) + n(\omega ,G,t)}{2} \, \left[ \tau (\omega ,G_\textrm{base}) - \tau (\omega ,G)\right] \,, \end{aligned}$$

in year *t* measures the travel time savings $\tau (\omega , G_\textrm{base}) - \tau (\omega ,G)$ of cyclists across all trip-cyclist-type combinations $\omega$ compared to the base network $G_\textrm{base}$, employing the rule-of-half approximation to account for induced demand^59^. Here, $\tau (\omega , G)$ and $\tau (\omega , G_\textrm{base})$ denote the travel time of cyclists and $n(\omega ,G,t)$ and $n(\omega , G_\textrm{base})$ denote the cycling demand in the current and base network, respectively. The factor $\zeta$ represents the value of time, converting the travel time savings into a monetary value.

(ii) The annual health benefits (HB),

9 $$\begin{aligned} \textrm{HB}(G, t)&= \sum _\omega \xi (\omega ) \left[ n(\omega , G, t) \, \ell (\omega , G) - n(\omega , G_\textrm{base}) \, \ell (\omega , G_\textrm{base}) \right] \,, \end{aligned}$$

in year *t* capture the value of increased distance cycled over all trip-cyclist-type combinations $\omega$, mainly due to induced demand. Here, $\ell (\omega , G)$ and $\ell (\omega , G_\textrm{base})$ denote the physical trip distances in the current and base network, respectively. The factor $\xi$ again represents the conversion factor of the health benefits to monetary value.

(iii) The total construction costs (CC),

10 $$\begin{aligned} \textrm{CC}(G)&= \sum _{s \in S_{G}} \textrm{cc}(s) \,, \end{aligned}$$

denote the total financial investment in the cycle superhighway extensions for all built segments $S_{G}$ in the graph *G*, where $\textrm{cc}(s)$ are the construction cost for the individual segment *s*.

(iv) The annual maintenance costs (MC),

11 $$\begin{aligned} \textrm{MC}(G)&= \sum _{s \in S_{G}} \textrm{mc}(s) \,, \end{aligned}$$

denote the required financial investment for maintaining the current cycle superhighway extensions for all built segments $S_{G}$ in the graph *G*, where $\textrm{mc}(s)$ are the annual maintenance cost for the individual segment *s*.

(v) Finally, the total scrap value (SV),

12 $$\begin{aligned} \textrm{SV}(t, G)&= \kappa (t) \sum _{s \in S_{G}} \,\textrm{cc}(s) \,, \end{aligned}$$

denotes the remaining value of the construction costs of the cycle superhighway extensions at time *t*. The factor $\kappa (t)$ captures the devaluation as a function of time.

The net present value of a sequence of networks $\{G(t)\}$ up to year *t* of the planning stage (including the newly built cycle superhighway extensions) is then given by the sum of the annual components up to year *t* plus the one-time contributions of construction cost and scrap value. Importantly, the net present value contribution of each year is weighted with the devaluation factor $\kappa (t)$. In total, we have

13 $$\begin{aligned}&\phantom {=} \textrm{NPV}(t, \{G(t)\}) \nonumber \\&= \left[ \sum _{t' = 2}^{t} \kappa (t') \, {\textrm{TB}}(G(t'), t') \right] \nonumber \\&+ \left[ \sum _{t' = 2}^{t} \kappa (t') \, \textrm{HB}(G(t'), t') \right] - \Bigg [ \textrm{CC}(G(t)) \Bigg ] - \left[ \sum _{t' = 2}^{t} \kappa (t') \, \textrm{MC}(G(t'-1)) \right] + \Bigg [ \textrm{SV}(t, G(t)) \Bigg ] \nonumber \\&= \left[ \sum _{t' = 2}^{t} \kappa (t') \, \sum _\omega \zeta (\omega )\,\frac{n(\omega , G_\textrm{base}) + n(\omega , G(t'), t')}{2} \, \Big (\tau (\omega , G_\textrm{base}) - \tau (\omega , G(t'))\Big ) \right] \nonumber \\&\phantom {=} + \left[ \sum _{t' = 2}^{t} \kappa (t') \sum _\omega \xi (\omega ) \Big (n(\omega , G(t'), t') \, \ell (\omega , G(t')) - n_\omega (G_\textrm{base}) \, \ell (\omega , G_\textrm{base}) \Big ) \right] \nonumber \\&\phantom {=} - \left[ \sum _{s \in S_{G(t)}} \textrm{cc}(s) \right] - \left[ \sum _{t' = 2}^{t} \kappa (t) \sum _{s \in S_{G(t'-1)}} \textrm{mc}(s) \right] + \left[ \kappa (T) \sum _{s \in S_{G(t)}} \textrm{cc}(s) \right] \,, \end{aligned}$$

where the summation starts in the second year, after the first planned extensions have been constructed in year $t=1$, where only construction and scrap value contribute to the net present value.

### Direct optimization of the cycle superhighway network

**Batched optimization** The direct optimization model from Paulsen & Rich (2024)^8^ iteratively finds the best cycle superhighway segments to be built by maximizing the predicted net present value at the end of the 50-year planning period. The model incorporates expected changes in travel time and health benefits, including those driven by increased demand, to identify the optimal combination of segments to be constructed each year. The objective function $\Delta \,\widetilde{\textrm{NPV}}$ approximating the gain in net present value, defined for all segments not yet included in the graph *G* at a given period *t*, is expressed as

14 $$\begin{aligned} \Delta \,\widetilde{\textrm{NPV}}(s,G,t)&= \left[ \sum _{t' = t+1}^{T} \kappa (t') \right] \, \Delta {\widetilde{\textrm{TB}}}(s,G,t)\, + \left[ \sum _{t' = t+1}^{T} \kappa (t') \right] \Delta \widetilde{\textrm{HB}}(s,G,t) - \kappa (t) \,\textrm{cc}(s) - \left[ \sum _{t' = t+1}^{T} \kappa (t')\right] \, \textrm{mc}(s) \,. \end{aligned}$$

Here, $\widetilde{\textrm{TB}}$ represents the linearized estimates of raw travel time benefits, approximated using the travel times of the network *G* before the current decision step and the fully expanded network, $G_\textrm{full}$:

15 $$\begin{aligned} \Delta {\widetilde{\textrm{TB}}}(s,G,t) = \sum _\omega \zeta (\omega )\,\frac{n(\omega ,G_\textrm{base}) + \tilde{n}(\omega ,s,G,t)}{2} \, \left[ \tau (\omega ,G) - \tilde{\tau }(\omega ,s,G) \right] \,, \end{aligned}$$

with $\tilde{\tau }(\omega ,s,G)$ defined as

16 $$\begin{aligned} \tilde{\tau }(\omega ,s,G)&= \tau (\omega ,G) - \Big (\tau (\omega ,G) - \tau (\omega ,G_\textrm{full})\Big ) \frac{d\left( \omega ,s,G_\textrm{full}\right) }{\sum _{s' \notin G}d\left( \omega ,s',G_\textrm{full}\right) } \,, \end{aligned}$$

and $$\tilde{n}(\omega ,s,G,t)$$ as

17 $$\begin{aligned} \tilde{n}(\omega ,s,G,t)&= \gamma (t)\,n(\omega ,\tilde{\tau }(\omega , s,G))\,. \end{aligned}$$

The linearized estimations of health benefits $\Delta \widetilde{\textrm{HB}}$ are similarly defined as

18 $$\begin{aligned} \Delta \widetilde{\textrm{HB}}(\omega ,s,G)&= \sum _\omega \xi (\omega ) \left[ \tilde{n}(\omega ,s, G, t) \, \tilde{\ell }(\omega ,s,G) - n(\omega , G_\textrm{base}) \, \ell (\omega , G_\textrm{base}) \right] \,, \end{aligned}$$

with

19 $$\begin{aligned} \tilde{\ell }(\omega ,s,G)&= \ell (\omega ,G) - \left( \ell (\omega , G) - \ell (\omega , G_\textrm{full})\right) \frac{d\left( \omega ,s,G_\textrm{full}\right) }{\sum _{s'\notin G}d\left( \omega ,s',G_\textrm{full}\right) }\,. \end{aligned}$$

The concept behind the linearization^7^ is that, for each $\omega$, the changes in travel times and distances resulting from upgrading the entire network can be attributed to the segments utilized in the full network, $G_\textrm{full}$. The individual weighting of each segments *s* (as represented by the fractions in Eq. (16), Eq. (17), and Eq. (19)) is proportional to the distance traveled on *s* in the full network $G_\textrm{full}$. If the denominator is zero, the numerator will also be zero. In this case, the fraction should be treated as a zero and thus does not influence $\tilde{\tau }$, $\tilde{n}$ or $\tilde{\ell }$.

Based on the predicted change $\Delta \,\widetilde{\textrm{NPV}}(s,G,t)$ in the net present value for each segment *s*, we formulate a binary integer program over the set of non-selected segments. The problem is solved using a standard solver^60^ to find the optimal set $S_t = \left\{ s^*_{t,1}, s^*_{t,2}, \ldots \right\}$ to be built in year *t*. This set of segments maximizes the expected change in net present value subject to the budget constraint in the current year *t*,

20 $$\begin{aligned} \sum _{s \,\in \, S(t)} \textrm{cc}(s) \le \sum _{t' = 1}^{t} b(t') \;- \sum _{s \,\in \, S_{< t}} \textrm{cc}(s) - \sum _{t'=2}^t \, \sum _{s \,\in \, S_{< t'}} \textrm{mc}(s) , \end{aligned}$$

where *b* denotes the (constant) annual budget and $S_{< t} = \bigcup _{t' = 1}^{t-1} S_{t'}$ denotes the set of cycle superhighway segments constructed up to but not including year *t*. The second sum on the right-hand-side thus describes the total construction cost of all segments built until and including year $t-1$, the third sum describes the cumulative maintenance cost for all constructed segments, e.g. in year *t* we pay the maintenance cost for all cycle superhighway segments constructed in year $t-1$ and before.

After each year *t*, we add the set $S_t$ to *G* and recompute the estimated net present value changes for each remaining segment, update the budget constraints and select the next set of segments to be constructed until the expected change in net present value is negative for all remaining segments. The update from year to year requires recomputing the routes for each $\omega$ in order to update the actual and estimated travel times and distances of the shortest paths for each $\omega$.

**Greedy optimization** We also analyze a simpler version of the optimization algorithm from Paulsen & Rich (2023)^7^. This version differs from the more advanced approach^8^ described above by selecting segments greedily based on an estimated net present value rate per construction cost $\Delta \tilde{R}$ under the assumption of constant demand (comparable to the advanced evaluation functions for the backward percolation approach, see below),

21 $$\begin{aligned} \Delta \,\tilde{\textrm{R}}(s,t)&= \frac{ \left[ \sum _{t' = t+1}^{T} \kappa (t') \right] \, \Delta \widetilde{\textrm{TB}}(s,G_\textrm{base},0)\, - \kappa (t) \,\textrm{cc}(s) - \left[ \sum _{t' = t+1}^{T} \kappa (t')\right] \, \textrm{mc}(s) }{ \kappa (t)\,\textrm{cc}(s)}\,. \end{aligned}$$

This greedy approach does not consider optimal bundling of segments within a period and entirely excludes health benefits, as they are considered negligible under the assumption of constant demand.

As before, construction costs contribute immediately, while travel time benefits and maintenance costs contribute only in the years following construction ($t' \ge t+1$). It is assumed that the contribution from each segment *s* can be isolated, independent of other segments. In contrast to the batched optimization approach introduced above, the simplified travel time benefit estimates are computed based only on the initial and final network state $G_\textrm{base}$ and $G_\textrm{full}$ without recomputing the routes or travel times of cyclists. The approximated travel time benefits associated with segment *s* is thus a simplified case of Eq. (15),

22 $$\begin{aligned} \Delta {\widetilde{\textrm{TB}}}(s,G_\textrm{base},0) = \sum _\omega n(\omega ,G_\textrm{base}) \, \left[ \tau (\omega ,G_\textrm{base}) - \tilde{\tau }(\omega ,s,G_\textrm{base}) \right] \,. \end{aligned}$$

Using the predicted change $\Delta R(s,t)$ in net present value per construction cost for each segment *s*, we greedily select the set $S_t = \left\{ s^*_{t,1}, s^*_{t,2}, \ldots \right\}$ of segments to be constructed in year *t*. This set maximizes the expected change in net present value, satisfying $\Delta \widetilde{\textrm{NPV}}(s^*_{t,1}) \ge \Delta \widetilde{\textrm{NPV}}(s^*_{t,2}) \ge \ldots$, while adhering to the same budget constraint as specified in the advanced model (Eq. (20)). After each year, we recompute the estimates of the net present value rates and select the next set of segments to be built until all cycle superhighway segments have been added to the network (after 47 years). The update from year to year is much faster compared to the advanced method, as only the discount factors $\kappa$ depend on *t*. However, evaluation of the network quality requires additional post-processing and computation of the routes and travel times of all cyclists.

### Dynamic backward percolation approach

The backward percolation algorithm starts from the full network and iteratively removes the least important cycle superhighway segment from the network. The general procedure is as follows:

#### Algorithm

1. Set up initial network state $G \leftarrow G_\textrm{full}$
  1. Read in base network $G_\textrm{base}$
  2. Add all buildable cycle superhighway segments $s \in S$
  3. Calculate the routes of all trips
2. Main loop, iterate until all buildable cycle superhighway segments have been removed and $G = G_\textrm{base}$.
  2.a Calculate the evaluation function *Q*(*s*) for each remaining segment $s \subset G$.
  2.b Select the least important remaining segment $s^*$ with $Q(s^*) \le Q(s)$ for all $s \subset G$.
  2.c Remove the segment from the network, $G \leftarrow G \setminus s^*$.
  2.d Recalculate the routes for all affected trips
3. Algorithm finished.

**Evaluation criteria** We use three different evaluation criteria *Q* to determine the least important cycle superhighway segment to be removed in each step of the algorithm:

(i) The normalized travel time penalty $Q_\text {pen}$introduced in the original backward percolation model^32^,
  23 $$\begin{aligned} Q_\textrm{pen}(s, G)&= \frac{\sum _\omega \sum _{e \in s} n_e(\omega , G) \, l_e \, c_e(\omega )}{\sum _{e \in s} l_e} \,. \end{aligned}$$
  The penalty $c_e(\omega ) = \frac{v_\textrm{csh}(\omega )}{v_e(\omega )}> 1$ describes the relative change in travel time for cyclists when the cycle superhighway is removed, assuming cyclists do not change their routes. Here $v_\textrm{csh}(\omega )$ denotes the speed of cyclists traveling in the cycle superhighway network, while $v_e(\omega )$ denotes the speed of cyclists traveling in the original base network (compare Tab. 3). This measure is proportional to the total travel time for all cyclists $n_e(\omega , G)$ using edges *e* of the segment *s*. For newly built edges, we take the penalty *c* as the penalty of a street edge to avoid infinite importance of segments.
(ii) The relative change $Q_\textrm{stat}$,
  24 $$\begin{aligned} Q_\textrm{stat}(s, G)&= \frac{\sum _\omega \sum _{e \in s} \zeta (\omega ) \frac{n(\omega , G_\textrm{base}) + n(\omega , G)}{2} \Delta \tau _e(\omega )}{\textrm{CC}(s)} \nonumber \\&\approx \frac{\Delta {\textrm{TB}}(s,G)}{\textrm{CC}(s)} \,, \end{aligned}$$
  represent the relative change in net present value, attributable to travel time reduction, relative to the required investment cost-under the assumption that demand and route choices remain unchanged when the segment is removed. Here, $\Delta \tau _e(\omega )> 0$ describes the change in travel time when the cycle superhighway segment is removed.
(iii) The relative change $Q_\textrm{dyn}$ in travel time benefits and health benefits,
  25 $$\begin{aligned} Q_\textrm{dyn}(s, G)&= \frac{1}{CC(s)} \, \sum _\omega \sum _{e \in s} \left[ \zeta (\omega ) \, \left( \beta \, n(\omega ,G)(P(\omega , G)-1) \, \frac{\tau (\omega , G_\textrm{base})-\tau (\omega , G)}{2} + \frac{n(\omega , G_\textrm{base})+n(\omega , G)}{2}\right) \right. \nonumber \\&\quad \quad \quad \quad \quad \quad \quad + \; \xi (\omega ) \, \beta \, n(\omega ,G) \, (P(\omega , G)-1) \, \ell (\omega , G) \bigg ] \, \Delta \tau _e(\omega ) \nonumber \\&\approx \frac{\Delta {\textrm{TB}}(s, G) + \Delta \textrm{HB}(s, G)}{\textrm{CC}(s)} \,, \end{aligned}$$
  reflects the relative change in net present value, driven by travel time reductions and induced demand, in proportion to the required investment costs, under the assumption of fixed route choices.

Here, we evaluate the demand $n(\omega , G)$ at time $t=0$ without population growth and neglect future maintenance costs, since the exact time of construction of the segment is unknown during the planning stage for the backward algorithm. Including population growth would only add a multiplicative factor to the importance of all segments without changing the relative ordering.

Details on the extension of the original evaluation function for individual edges to the segment-evaluation $Q_\textrm{pen}$ are given in Supplementary Note 1. Derivations of the evaluation criteria $Q_\textrm{stat}$ and $Q_\textrm{dyn}$ from the net present value are given in Supplementary Note 3.

## Supplementary Information

Supplementary Information.

## Data Availability

Data will be made available on request to M.P.

## Code availability

The code for the backwards percolation algorithm is available through GitHub (https://github.com/PhysicsOfMobility/BikePathNet) under AGPL-3.0 license. The specific version of the package used to produce the results for this manuscript is available from Zenodo^61^. Code for the greedy direct optimization and the evaluations of the net present value is also available through GitHub (https://github.com/madspDTU/OptimalBicycleExpansion). Code for the batched direct optimization is available upon request.
