# Supplementary material for: Robust design of bicycle infrastructure networks
Source: Sci Rep. 2025 May 3;15:15471. doi: 10.1038/s41598-025-99976-9 (PMC12048686; doi:10.1038/s41598-025-99976-9)
Supplement: Supplementary file 1 — Supplementary Information. [file 41598_2025_99976_MOESM1_ESM.zip › RobustDesignOfBicycleInfrastructureNetworks_Supp.pdf]

Supplemental Material

accompanying the Manuscript

Robust design of bicycle infrastructure networks

Christoph Steinacker[1,*], Mads Paulsen[2], Malte Schröder[1,+], and Jeppe Rich[2,+]

[1]Chair of Network Dynamics, Center for Advancing Electronics Dresden (cfaed) and Institute of Theoretical Physics, TUD Dresden University of Technology, 01062 Dresden, Germany
[2]Transportation Science Division, Department of Technology, Management and Economics, Technical University of Denmark, 2800 Kgs. Lyngby, Denmark
[*]christoph.steinacker@tu-dresden.de
[+]these authors contributed equally to this work

Supplementary Note 1: Bikeability with variable demand

In the original proof-of-concept introduction of the dynamic backwards percolation approach[1], the quality of bike path networks G was evaluated with the bikeability,

$$B(G) = \frac{T(G_{\text{base}}) - T(G)}{T(G_{\text{base}}) - T(G_{\text{full}})} \tag{S1}$$

based on the total effective travel time in the network

$$T(G) = \sum_{\omega} T(\omega, G) = \sum_{\omega} n(\omega, G)\, \tau(\omega, G) \tag{S2}$$

compared to the maximum possible travel time $T(G_{\text{base}})$ in the base state and the minimum possible travel time $T(G_{\text{full}})$ in the fully built state of the network. The bikeability describes the normalized change between the two extreme states, thus offering a transferable measure for the improvement of cycling compared to its maximum potential (i.e. without discriminating against cities where cycling is difficult by default, for example due to the geography or topography).

However, with variable demand, the total travel time is not a suitable measure. The total travel time $T(\omega) = n(\omega, G)\, \tau(\omega, G)$ for one trip ω may increase even though the travel time $\tau(\omega, G)$ for a trip decreases due to induced demand increasing the number of cyclists $n(\omega, G)$. Note that we here only consider induced demand due to shorter travel times, not demand changes due to population growth, such that we work without explicit reference to time in the following.

We define a new loss function L, explicitly taking into account the variable demand $n(\omega, \tau)$ as a function of the effective travel time $\tau(\omega, G)$ of each trip ω. As described in the Methods section in the main manuscript, the demand curve for each trip ω is given as a logit model of the form

$$n(\omega, \tau) = n(\omega)\, P(\omega, \tau) = n(\omega) \frac{e^{\beta\, \tau}}{e^{\beta\, \tau} + e^{\beta\, \tau_{\text{other}}(\omega)}}\,, \tag{S3}$$

where we substitute the implicit dependence on the network G with the explicit dependence on the travel time τ to simplify the calculations here and in Supplementary Note 3. The change in loss ΔL for a single trip ω should be given by the change in travel time, $\Delta L(\omega) = \Delta T(\omega) = n(\omega, \tau)\, \Delta\tau$ for a change in the network causing a change $\Delta\tau$ of the travel time. Additional effects like induced demand introduce only higher order corrections proportional to $\Delta\tau^2$ that we neglect here. With this condition, the evaluation function defined in Supplementary Note 2 [Eq. (S13)] estimates this difference relative to the length of the segments, as in the original definition. For a single trip ω, we thus have a loss function of the form

$$L(\omega, G) = \int_0^{\tau(\omega,G)} n(\omega, \tau')\, \mathrm{d}\tau' \tag{S4}$$

$$= n(\omega) \int_0^{\tau(\omega,G)} \frac{e^{\beta\, \tau'}}{e^{\beta\, \tau'} + e^{\beta\, \tau_{\text{other}}(\omega)}}\, \mathrm{d}\tau' \tag{S5}$$

$$= \frac{n(\omega)}{\beta} \ln\left(\frac{e^{\beta\, \tau(\omega,G)} + e^{\beta\, \tau_{\text{other}}(\omega)}}{1 + e^{\beta\, \tau_{\text{other}}(\omega)}} \right). \tag{S6}$$

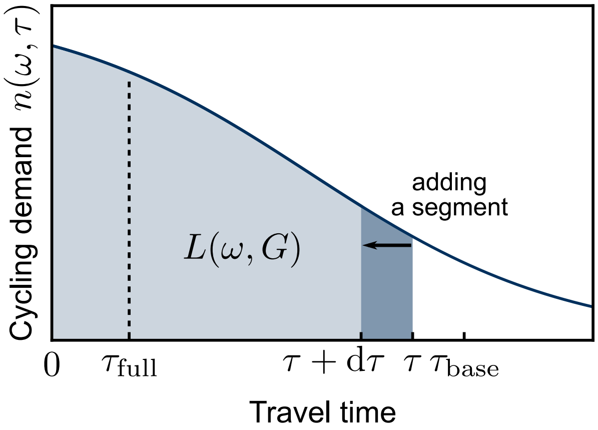

Figure S1. Bikeability loss function with variable demand. The loss function $L(\omega,G)$ describes the area under the demand curve (shaded area). A reduction in travel time due to an added segment (black arrow, dark blue area) reduces the loss function. In the limit of constant demand $n(\omega)$, the loss function becomes $L(\omega,G) = n(\omega)\,\tau(\omega,G) = T(\omega,G)$, equal to the total travel time of cyclists. The bikeability [Eq. (S11)] evaluates the relative change of the loss function between the base network (large travel times τ_{base}) and the fully upgraded network (small travel times τ_{full}).

This loss function measures the area under the demand curve $n(\tau)$ (Fig. S1). When the travel time τ becomes smaller, $d\tau < 0$, the loss reduces with $dL(\omega) = n(\omega,\tau)\,d\tau < 0$ as required. The same travel time reduction $d\tau$ has a larger effect if the travel time is already small and the number of cyclists is large. For static demand $n(\omega,\tau) = n_{\text{base}}(\omega) = n(\omega,\tau(\omega,G_{\text{base}}))$, this definition simplifies to the original function

$$
\begin{aligned}
L(\omega,G) &= \int_0^{\tau(\omega,G)} n_{\text{base}}(\omega)\,d\tau' && \text{(S7)}\\
&= n_{\text{base}}(\omega)\,\tau(\omega,G) && \text{(S8)}\\
&= T(\omega,G)\,, && \text{(S9)}
\end{aligned}
$$

measuring the total travel time.

For multiple trips, the total loss function of the network is simply the sum of the loss over all trips,

$$
L(G) = \sum_{\omega} L(\omega,G)\,. \tag{S10}
$$

To compute the overall bikeability of the network, we employ the same idea of comparing the loss function of the network with the initial and the fully built network,

$$
B(G) = \frac{L(G_{\text{base}}) - L(G)}{L(G_{\text{base}}) - L(G_{\text{full}})}\,. \tag{S11}
$$

The bikeability $B(G) = 0$ in the initial network and increases as more bike paths are built, up to $B(G) = 1$ in the fully upgraded network [Eq. (4) in the main manuscript].

Supplementary Note 2: Consistent evaluation of bike link importance

In the original proof-of-concept introduction of the dynamic backwards percolation approach[1], the importance of a single edge e in the bike path network was estimated by the product of the number of cyclists n_e using that edge and the penalty $c_e = \frac{v_{\text{csh}}}{v_e} > 1$ on the travel time of the edge when the bike path was removed, where v_{csh} and v_e denote the speed of cyclists on the cycle superhighway edge and the base network edge, respectively. Together, the importance measure is

$$Q_{\text{pen}}(e) = n_e\, c_e = \frac{n_e\, c_e\, l_e}{l_e}\,. \tag{S12}$$

This measure is proportional to the total travel time $T_e = n_e\, c_e\, \frac{l_e}{v_{e,0}}$ on the edge per unit length of the edge.

However, the cycle superhighway network in the main manuscript consists of segments which each include multiple edges. We now define an evaluation function across multiple edges at the same time to extend the penalty-based evaluation. For consistency, the evaluation function should fulfill two general conditions:

(i) a segment consisting of multiple identical edges with the same number of cyclists should be assigned the same importance as the individual edges

(ii) a segment consisting of a single edge should be assigned the same importance as the individual edge

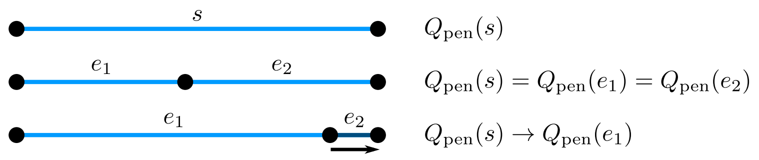

Figure S2. Consistent definition of segment importance. The definition of importance for edges and segments consisting of multiple edges should fulfil consistency conditions such that the resulting planned networks do not depend on the formatting of input data. In particular, the importance of an edge or a segment (top) should not change if it is split into multiple edges (middle) and should not be affected by edges with negligible length (bottom).

Condition (i) ensures that the order in which edges are removed does not depend on the fine-graining of edges in the network. For example, splitting edges and adding additional nodes or merging edges in a segment to a single edge should not change the importance. This condition also already ensured that the original definition was independent of the fine-graining of the network. Condition (ii) ensures that the definition for segments is consistent with the original evaluation function in the limiting case of a segment consisting of a single edge.

These two conditions are fulfilled by a length-weighted sum of the contribution of the individual edges in a segment [Eq. (17) in the main manuscript],

$$Q_{\mathrm{pen}}(s,G) = \frac{\sum_{\omega}\sum_{e\in s} n_e(\omega,G)\, l_e\, c_e(\omega)}{\sum_{e\in s} l_e}\,. \tag{S13}$$

where the travel time penalty now depends on the infrastructure type of the edge and the cyclist type of the trip.

This evaluation function again describes the total added travel time ΔT along all edges of the segment relative to the total length of the segment and thus naturally reduces to the original version when considering only a single edge. Here, we evaluate the importance of the segment with respect to the demand $n(\omega,G)$ in the current network, thus ignoring any additional induced demand due to already built bike paths.

To check the first condition, consider n cyclists from a single trip ω crossing a segment $s=(e_1,e_2)$ with two identical edges, $c_1=c_2=c$, with arbitrary length. The measure is identical to the original definition for a single edge,

$$Q_{\mathrm{pen}}(s) = \frac{n\, l_1\, c_1 + n\, l_2\, c_2}{l_1+l_2} \tag{S14}$$

$$= \frac{n\, c\, (l_1+l_2)}{l_1+l_2} \tag{S15}$$

$$= n\, c \tag{S16}$$

$$= Q_{\mathrm{pen}}(e_1) = Q_{\mathrm{pen}}(e_2) \tag{S17}$$

since the length of the segment cancels out due to the identical parameters.

To explicitly check the second condition, consider n cyclists from a single trip ω crossing a segment $s=(e_1,e_2)$, such that the number of cyclists on both edges is the same $n_1=n_2=n$. Here, the two edges may be different, $c_1\neq c_2$, but we assume that the second edge has negligible length, $l_2\to 0$. Then, the measure reduces to the original definition on the first edge

$$Q_{\mathrm{pen}}(s) = \lim_{l_2\to 0} \frac{n\, l_1\, c_1 + n\, l_2\, c_2}{l_1+l_2} \tag{S18}$$

$$= \frac{n\, c_1\, l_1}{l_1} \tag{S19}$$

$$= n_1\, c_1 \tag{S20}$$

$$= Q_{\mathrm{pen}}(e_1) \tag{S21}$$

since the second edge does not contribute to the importance weighted by its length.

Supplementary Note 3: Derivation of the evaluation criteria for the dynamic backward percolation approach

To achieve a fairer comparison to the direct optimization approach, we derive two additional evaluation functions for the backward percolation approach explicitly based on the net present value

$$\mathrm{NPV}(t) = \mathrm{TB}(t) + \mathrm{HB}(t) - \mathrm{CC}(t) - \mathrm{MC}(t) + \mathrm{SV}(t)\,. \tag{S22}$$

As discussed in the main manuscript, the positive effects of the travel time benefits TB, and the health benefits HB dominate the net present value. Moreover, construction and maintenance costs and scrap value depend explicitly on the timing of construction, not just the ordering of construction. They are thus difficult to estimate in the backward percolation approach. Therefore, we focus on estimating the effects of individual cycle superhighway segments on the travel time benefits and the health benefits.

The travel time benefits describe the value of changes in the travel time τ due to new bike paths, computed with the rule-of-half to account for the variable demand. The health benefits describe the value of the distance ℓ travelled by all cyclists (compare Methods section in the main manuscript). We thus start from the definitions

$$\mathrm{TB}(t) = \sum_{\omega} \frac{1}{2} \zeta(\omega) \left[n_{\mathrm{base}}(\omega) + n(\omega,\tau)\right] \left[\tau_{\mathrm{base}}(\omega) - \tau(\omega)\right] \tag{S23}$$

$$\mathrm{HB}(t) = \sum_{\omega} \xi(\omega) \left[n(\omega,\tau)\ell(\omega) - n_{\mathrm{base}}(\omega)\ell_0(\omega)\right]\,. \tag{S24}$$

where $n(\omega,\tau)$ and $n_{\mathrm{base}}(\omega) = n(\omega,\tau_{\mathrm{base}})$ denote the number of cyclists for a trip-cyclist type-combination ω and $\tau(\omega)$ and $\tau_{\mathrm{base}}(\omega)$ denote their travel time in the current network G and the initial network G_{base}, respectively.

In general, removing any segment s of the cycle superhighway network affects the travel time benefits and the health benefits by changing the travel time τ, the travel distance ℓ, and the number of cyclists n. Expanding the changes $\Delta\mathrm{TB}$ and $\Delta\mathrm{HB}$ in these variables yields

$$\Delta\mathrm{TB} = \mathrm{TB}(\text{current state}) - \mathrm{TB}(\text{edge removed}) \tag{S25}$$

$$= \sum_{\omega} \frac{1}{2} \zeta(\omega) \left[\left(n_{\mathrm{base}}(\omega) + n(\omega,\tau)\right)\left(\tau_{\mathrm{base}}(\omega) - \tau(\omega)\right) - \left(n_{\mathrm{base}}(\omega) + n(\omega,\tau'(\omega))\right)\left(\tau_{\mathrm{base}}(\omega) - \tau'(\omega)\right)\right] \tag{S26}$$

$$= \sum_{\omega} \frac{1}{2} \zeta(\omega) \left[-\left(\tau_{\mathrm{base}}(\omega) - \tau(\omega)\right)\Delta n(\omega,\tau) + \left(n_{\mathrm{base}}(\omega) + n(\omega,\tau)\right)\Delta\tau(\omega) + \Delta n(\omega,\tau)\Delta\tau(\omega)\right], \tag{S27}$$

$$\Delta\mathrm{HB} = \mathrm{HB}(\text{current state}) - \mathrm{HB}(\text{edge removed}) \tag{S28}$$

$$= \sum_{\omega} \xi(\omega) \left[\left(n(\omega,\tau)\ell(\omega) - n_{\mathrm{base}}(\omega)\ell_{\mathrm{base}}(\omega)\right) - \left(n(\omega,\tau'(\omega))\ell'(\omega) - n_{\mathrm{base}}(\omega)\ell_{\mathrm{base}}(\omega)\right)\right] \tag{S29}$$

$$= \sum_{\omega} \xi(\omega) \left[n(\omega,\tau)\ell(\omega) - \left(n(\omega,t) + \Delta n(\omega,\tau)\right)\left(\ell(\omega) + \Delta\ell(\omega)\right)\right] \tag{S30}$$

$$= \sum_{\omega} \xi(\omega) \left[-n(\omega,\tau)\Delta\ell(\omega) - \Delta n(\omega,\tau)\ell(\omega) - \Delta n(\omega,t)\Delta\ell(\omega)\right] \tag{S31}$$

where the dashed variables denote the values for the network $G \setminus s$ in which the segment is removed, $n(\omega,\tau') = n(\omega,\tau) + \Delta n(\omega,\tau)$, $\tau'(\omega) = \tau(\omega) + \Delta\tau(\omega)$, and $\ell'(\omega) = \ell(\omega) + \Delta\ell$.

We assume that cyclists do not change their route as a result of the change in the network,

$$\Delta\ell = 0\,. \tag{S32}$$

This simplification is the same as for the original evaluation function[1] and strongly reduces computation times by avoiding recalculation of routes to evaluate the importance of each segment in the network in every step. The change in travel time $\Delta\tau(\omega)$ is then given by the sum of all changes in travel time along each edge included in the highway segment s

$$\Delta\tau(\omega) = \sum_{e\in s} \Delta\tau_e(\omega) = \sum_{e\in s} \frac{l_e}{v_e(\omega)} - \frac{l_e}{v_{\mathrm{csh}}(\omega)} = \frac{l_e}{v_{\mathrm{csh}}(\omega)} \Delta c_e(\omega)\,, \tag{S33}$$

where l_e is the length of edge e and $\Delta c_e(\omega) = c_e(\omega) - 1 = \frac{v_{\mathrm{csh}}(\omega)}{v_e(\omega)} - 1$ is the change of the travel time penalty on the edge. Here, again, $v_{\mathrm{csh}}(\omega)$ denotes the speed of cyclists on a cycle superhighway and $v_e(\omega)$ denotes the speed of cyclists on the base

infrastructure type of the edge. Finally, the number $n(\omega,\tau)$ of cyclists is not an independent function but depends on the travel time $\tau(\omega)$. We thus express the change in demand as a function of the travel time reduction, linearizing the expression

$$\begin{aligned}
\Delta n(\omega,\tau) &= n(\omega,\tau') - n(\omega,\tau) && \text{(S34)}\\
&= n(\omega)\,P(\omega,\tau') - n(\omega)\,P(\omega,\tau) && \text{(S35)}\\
&= n(\omega)\left(\frac{e^{\beta\,\tau'}}{e^{\beta\,\tau'}+e^{\beta\,\tau_{\text{other}}}} - \frac{e^{\beta\,\tau}}{e^{\beta\,\tau}+e^{\beta\,\tau_{\text{other}}}}\right) && \text{(S36)}\\
&= n(\omega)\left(\frac{e^{\beta\,\tau+\Delta\tau}}{e^{\beta\,\tau+\Delta\tau}+e^{\beta\,\tau_{\text{other}}}} - \frac{e^{\beta\,\tau}}{e^{\beta\,\tau}+e^{\beta\,\tau_{\text{other}}}}\right) && \text{(S37)}\\
&\approx n(\omega)\left(\frac{e^{\beta\,\tau}}{e^{\beta\,\tau}+e^{\beta\,\tau_{\text{other}}}} + \left(\frac{\beta e^{\beta\,\tau}}{e^{\beta\,\tau}+e^{\beta\,\tau_{\text{other}}}} - \frac{\beta\left(e^{\beta\,\tau}\right)^2}{\left(e^{\beta\,\tau}+e^{\beta\,\tau_{\text{other}}}\right)^2}\right)\Delta\tau(\omega) - \frac{e^{\beta\,\tau}}{e^{\beta\,\tau}+e^{\beta\,\tau_{\text{other}}}}\right) && \text{(S38)}\\
&= n(\omega)\left(\beta\frac{e^{\beta\,\tau}}{e^{\beta\,\tau}+e^{\beta\,\tau_{\text{other}}}} - \beta\left(\frac{e^{\beta\,\tau}}{e^{\beta\,\tau}+e^{\beta\,\tau_{\text{other}}}}\right)^2\right)\Delta\tau(\omega) && \text{(S39)}\\
&= \beta\,n(\omega)\left(P(\omega,\tau) - P^2(\omega,\tau)\right)\Delta\tau(\omega) && \text{(S40)}\\
&= \beta n(\omega,\tau)\,(1-P(\omega,\tau))\,\Delta\tau(\omega) && \text{(S41)}\\
&= \sum_{e\in s}\beta n(\omega,\tau)\,(1-P(\omega,\tau))\,\Delta\tau_e(\omega)\,, && \text{(S42)}
\end{aligned}$$

where the last line gives the expression for a segment s as the sum over all edges e in the segment.

We now define the evaluation function Q_{dyn} as the linearized change in travel time benefits and health benefits relative to the construction costs of the segment,

$$\begin{aligned}
Q_{\text{dyn}}(s) &\approx \frac{\Delta\text{TB}(s)+\Delta\text{HB}(s)}{CC(s)} && \text{(S43)}\\
&\approx \frac{\sum_\omega \frac{1}{2}\zeta(\omega)\left[-\left(\tau_{\text{base}}(\omega)-\tau(\omega)\right)\Delta n(\omega,\tau) + \left(n_{\text{base}}(\omega)+n(\omega,\tau)\right)\Delta\tau(\omega)\right] + \xi(\omega)\left[-\Delta n(\omega,\tau)\ell(\omega)\right]}{CC(s)} && \text{(S44)}\\
Q_{\text{dyn}}(s) &= \frac{1}{CC(s)}\sum_\omega\sum_{e\in s}\Bigg[\zeta(\omega)\left(\beta n(\omega,t)\,(P(\omega,\tau)-1)\,\frac{\tau_{\text{base}}-\tau(\omega)}{2} + \frac{n_{\text{base}}+n(\omega,\tau)}{2}\right)\\
&\qquad + \xi(\omega)\beta n(\omega,t)(P(\omega,\tau)-1)\ell(\omega)\Bigg]\Delta\tau_e(\omega)\,. && \text{(S45)}
\end{aligned}$$

As a further simplification, we assume locally static demand, setting $\Delta n(\omega,t)=0$ but still evaluating all expression with the current, variable demand. Only the middle term in the evaluation function Eq. (S45) remains, giving

$$\begin{aligned}
Q_{\text{stat}} &\approx \frac{\Delta\text{TB}(s)}{CC(s)} && \text{(S46)}\\
&\approx \frac{\sum_\omega \zeta(\omega)\frac{n_{\text{base}}(\omega)+n(\omega,\tau)}{2}\Delta\tau(\omega)}{CC(s)} && \text{(S47)}\\
Q_{\text{stat}} &= \frac{\sum_\omega\sum_{e\in s}\zeta(\omega)\frac{n_{\text{base}}(\omega)+n(\omega,\tau)}{2}\Delta\tau_e(\omega)}{CC(s)}\,. && \text{(S48)}
\end{aligned}$$

Supplementary Note 4: Performance overview

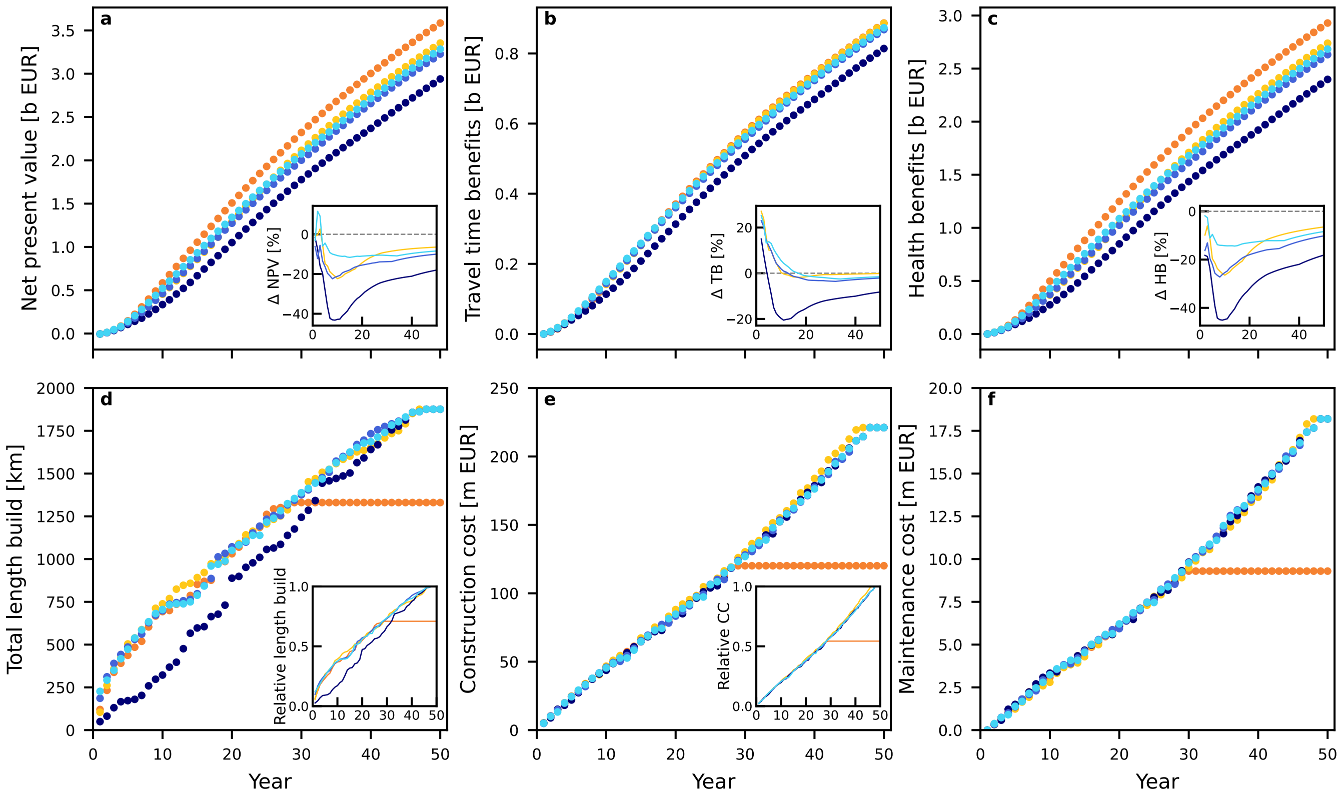

Figure S3. Performance overview for all five approaches. (a) Net present value (b) travel time benefits (TB) and (c) health benefits (HB) with (insets a-c) the relative difference to the batched optimization. (d) Total length build with relative length build in the inset, (e) total construction costs (CC) with relative construction costs in the inset and (f) maintenance costs per year. Net present value, travel time benefits, and health benefits are calculated as defined in the main manuscript (compare Eq. 13, Eq. 8, and Eq. 9, respectively). The construction and maintenance costs are only the simple sum of the costs of the built segments so far, discarding any discount factors.

Supplementary Note 5: Robustness of planned network expansion

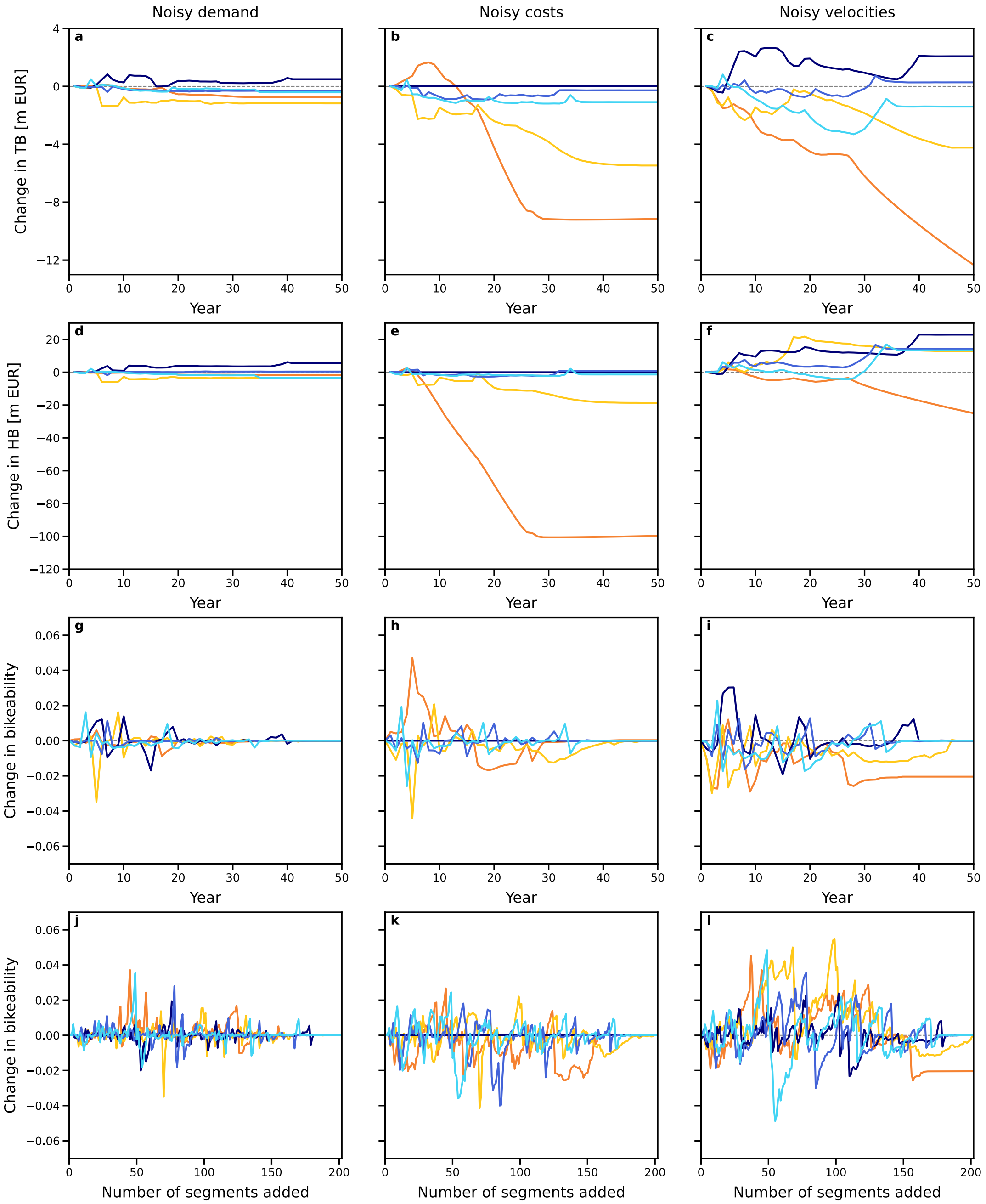

Figure S4. Changes of network quality measures under noisy input. Results for network quality measures [travel time benefits (top), bikeability by year (center), bikeability by segment (bottom)] to noise demand input (left), noisy segment costs (middle), and noisy velocities (right) are qualitatively similar to the results for the net present value (compare Fig. 7 in the main manuscript). Each line shows the average change over 10 realization compared to exact input data.

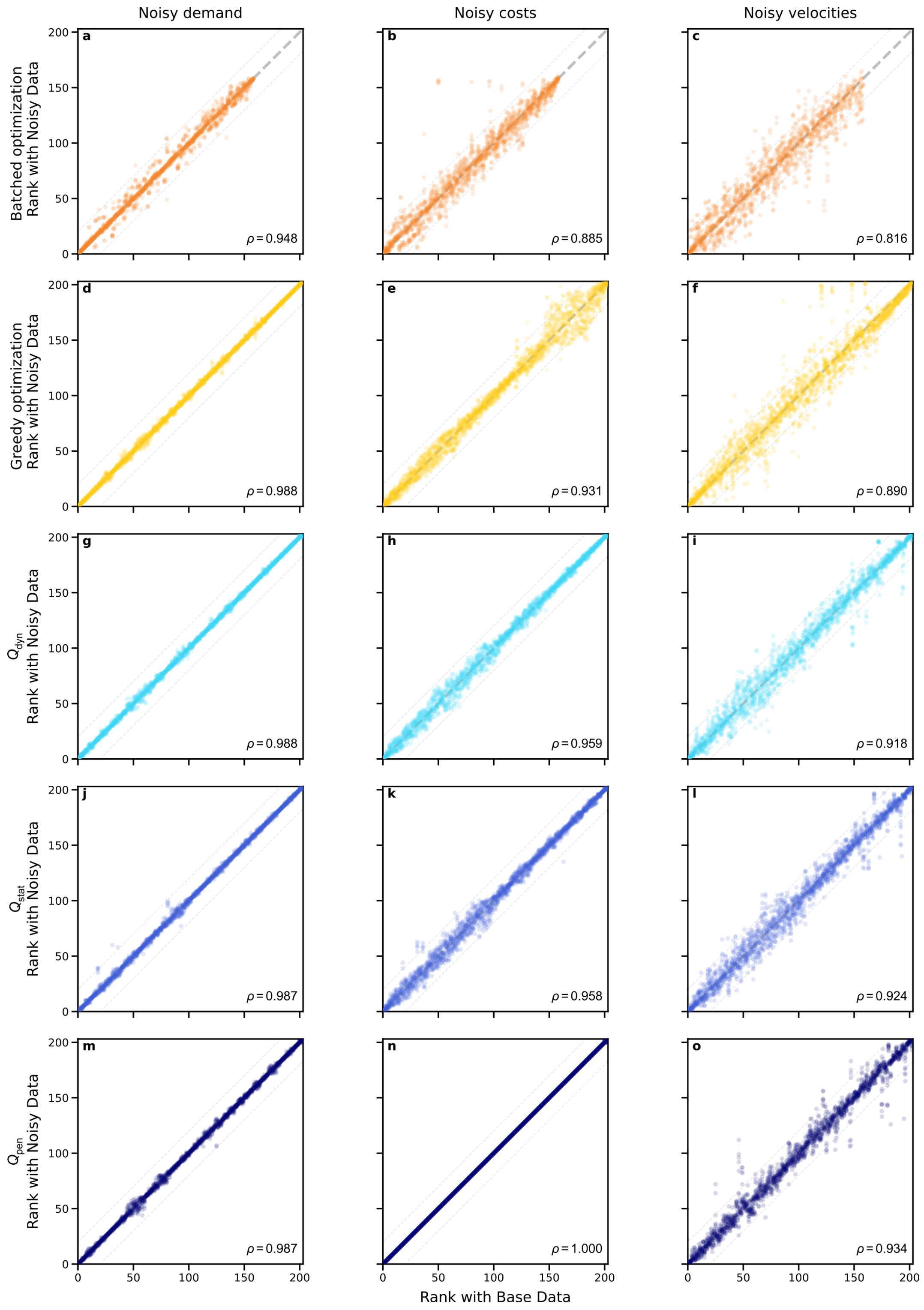

Figure S5. Rank differences of segment construction under noisy input data. Data on the identity indicates no change in rank. Kendall rank correlation coefficients ρ (bottom right in each panel) confirm the qualitative differences observed in the main manuscript. Complex evaluation functions [(greedy) direction optimization (top) or Q_{dyn} (middle)] consistently have a larger impact on the segment ordering, whereas simpler evaluation functions [Q_{stat} and Q_{pen} (bottom)] tend to be more robust. Noisy global parameters like velocities have a larger impact than noisy distributed parameters like demand, where effects may average out over the network. The panels show rank data for 10 realizations of noisy input each.

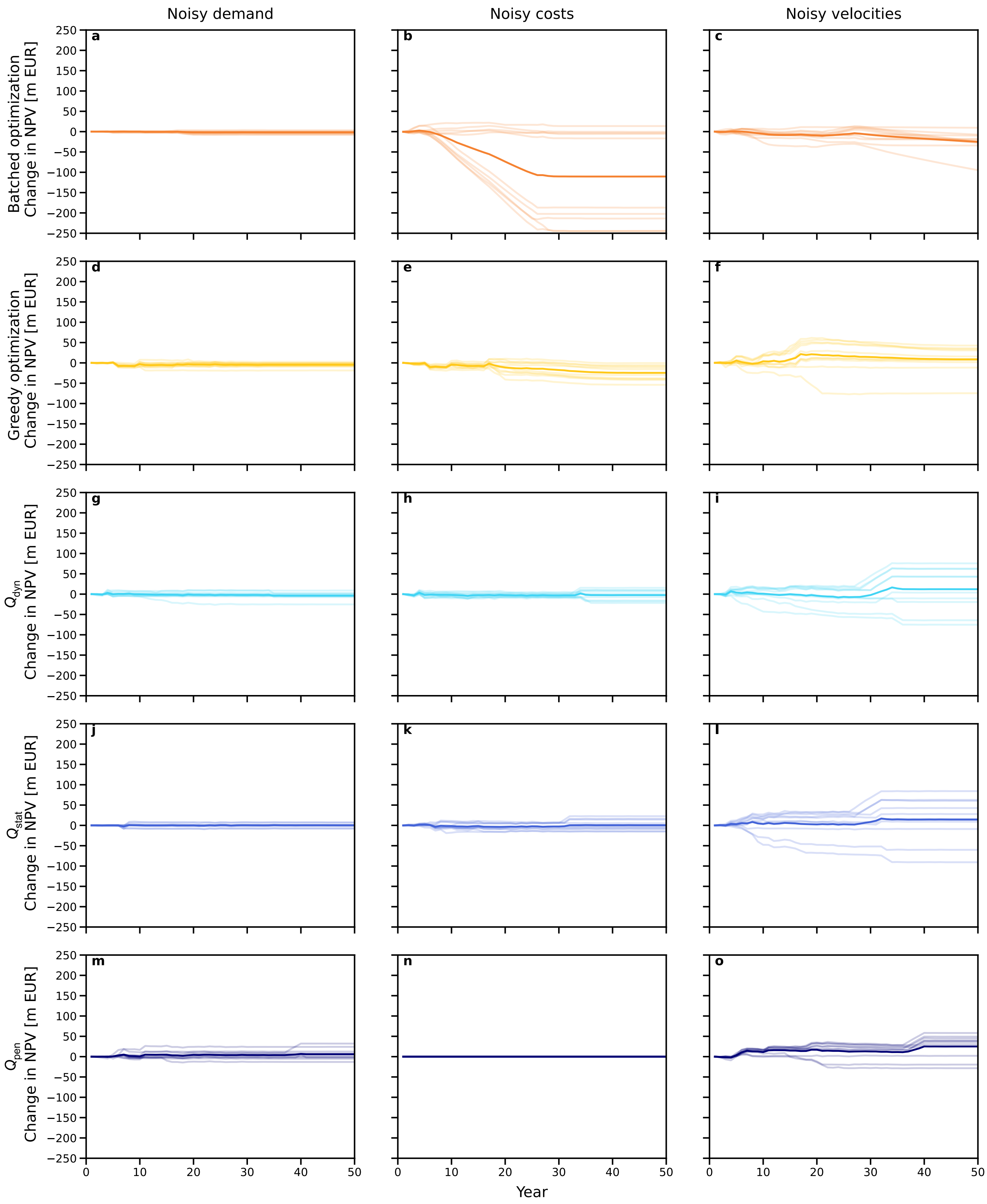

Figure S6. Change in net present value under noisy input data. Each panel shows 10 independent realization (light lines) and the average (dark line).

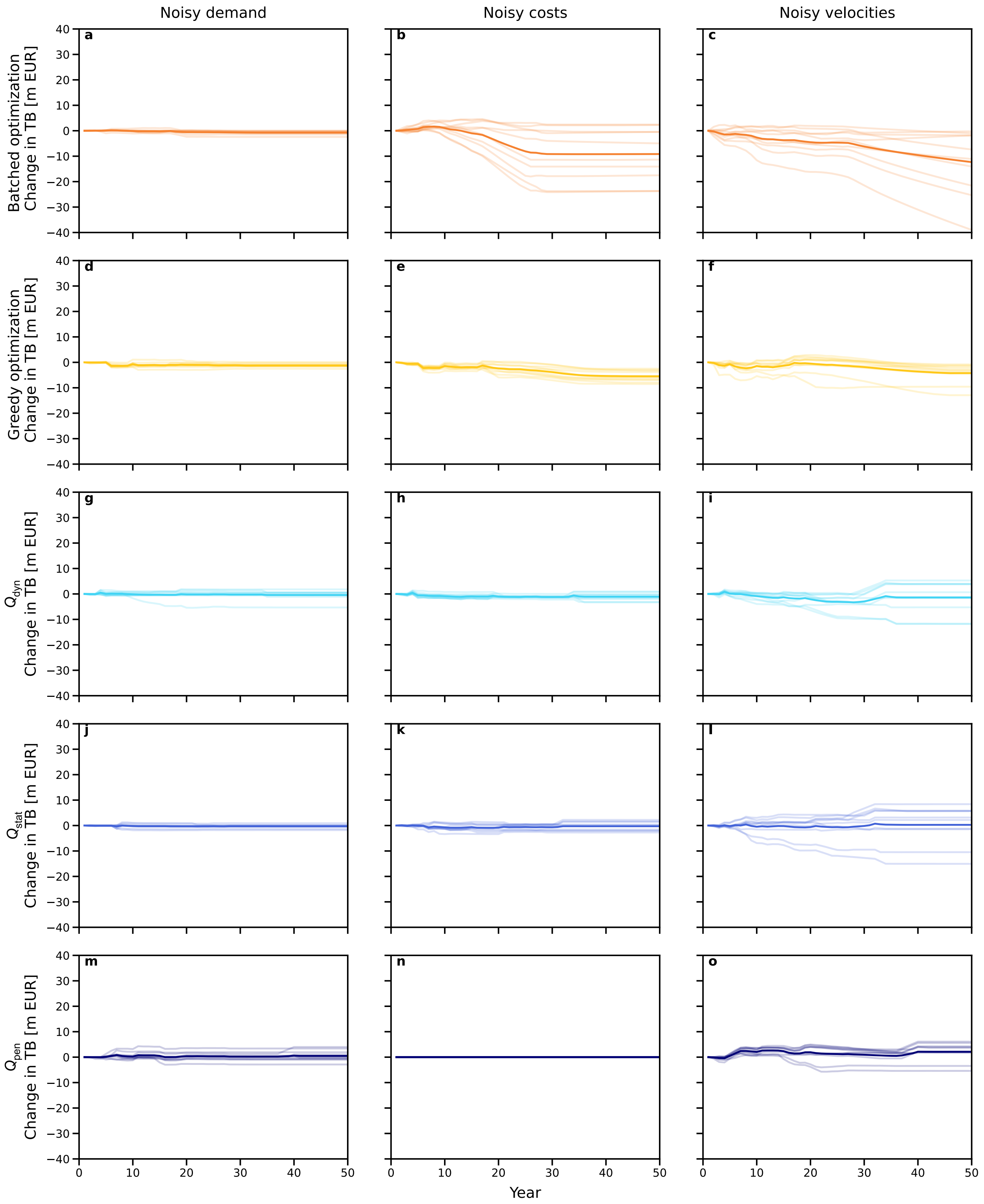

Figure S7. Change in travel time benefits under noisy input data. Each panel shows 10 independent realization (light lines) and the average (dark line).

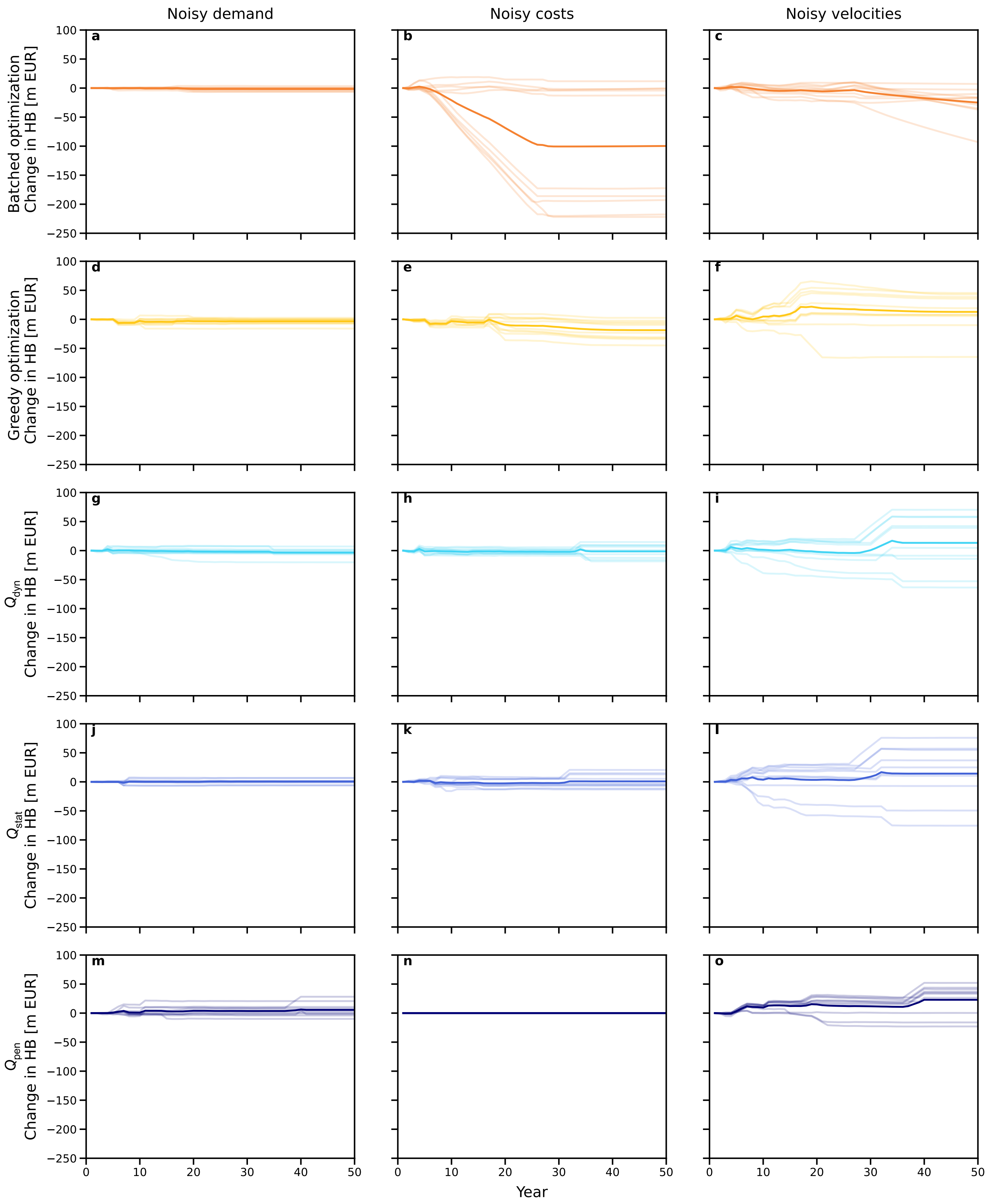

Figure S8. Change in health benefits under noisy input data. Each panel shows 10 independent realization (light lines) and the average (dark line).

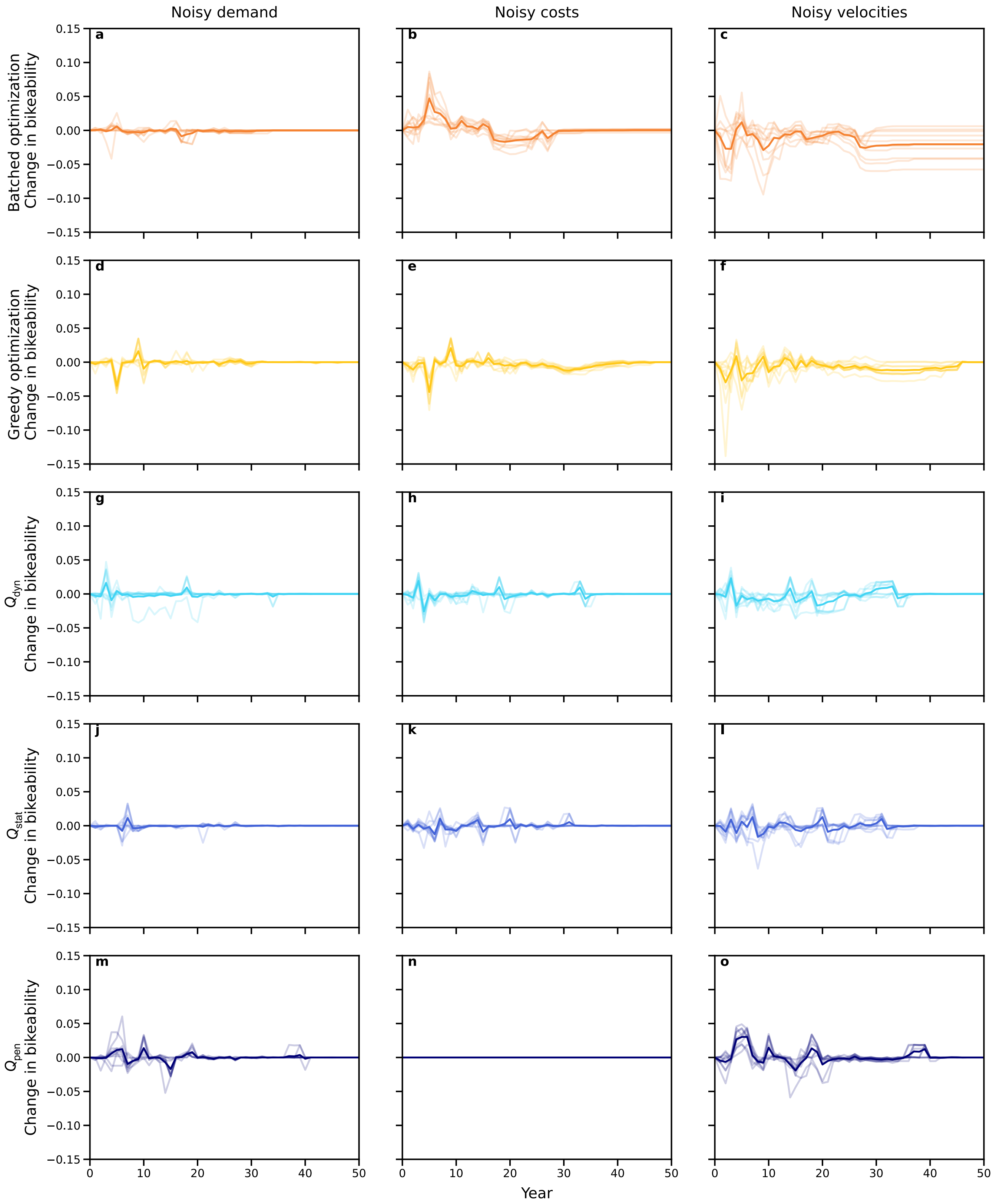

Figure S9. Change in bikeability per year under noisy input data. Each panel shows 10 independent realization (light lines) and the average (dark line).

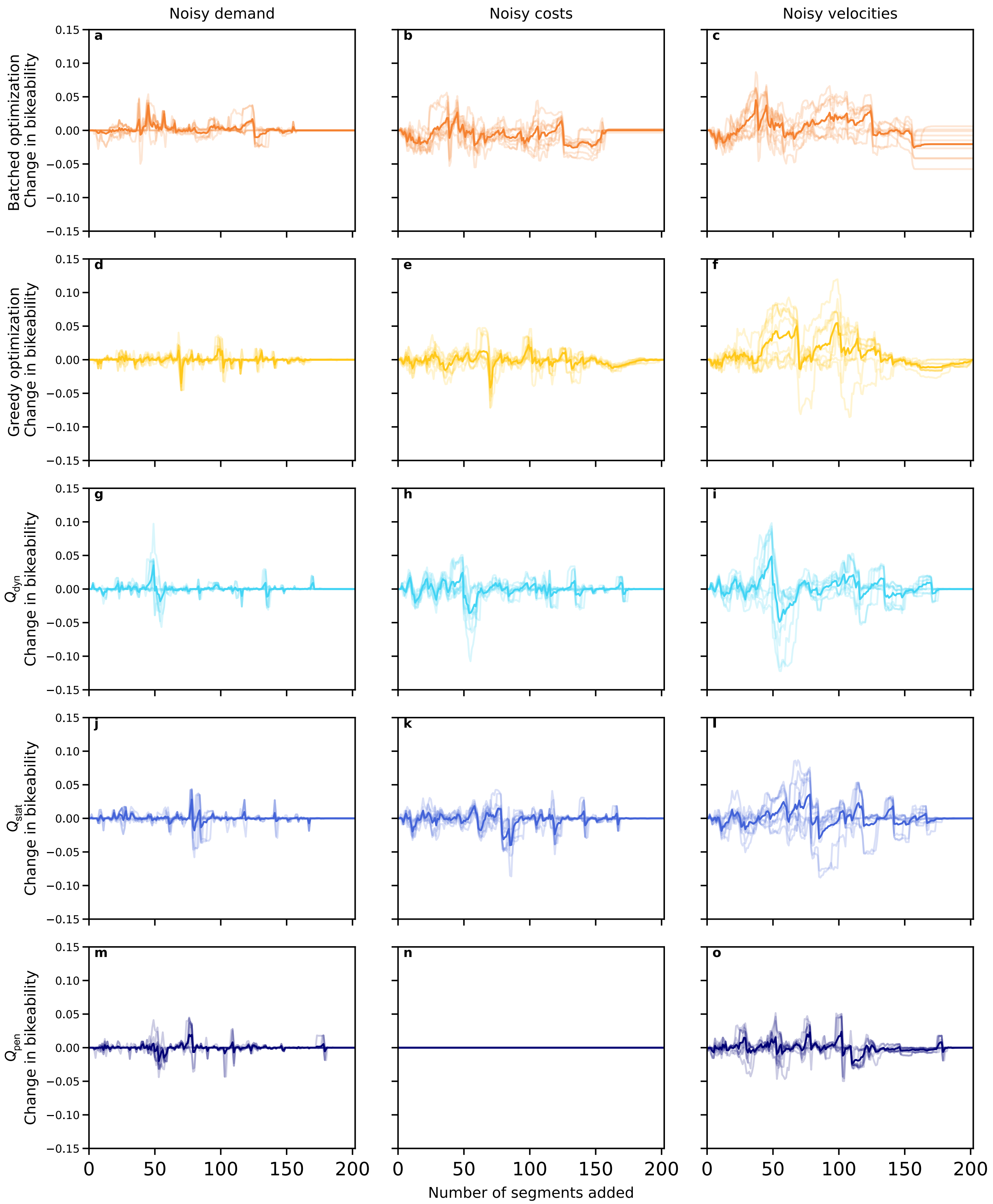

Figure S10. Change in bikeability per segment under noisy input data. Each panel shows 10 independent realization (light lines) and the average (dark line).

References

1. Steinacker, C., Storch, D.-M., Timme, M. & Schröder, M. Demand-driven design of bicycle infrastructure networks for improved urban bikeability. *Nat. Comput. Sci.* **2**, 655–664, DOI: 10.1038/s43588-022-00318-w (2022).
