# Supplementary material for: Robust design of bicycle infrastructure networks
Source: Sci Rep. 2025 May 3;15:15471. doi: 10.1038/s41598-025-99976-9 (PMC12048686; doi:10.1038/s41598-025-99976-9)
Supplement: Supplementary file 1 — Supplementary Information. [file 41598_2025_99976_MOESM1_ESM.zip › RobustDesignOfBicycleInfrastructureNetworks_SuppVideos_TitlesLegends.pdf]

Supp_Video_BP_Qdyn.mp4

Title: Evolution of planned network over 50 years by backward percolation with the dynamic-demand evaluation function Q_{dyn}.

Legend: Upgraded segment by Q_{dyn} (light blue), upgradable segments (beige), existing segments (dark gray), network (light gray)

Supp_Video_BP_ Qstat.mp4

Title: Evolution of planned network over 50 years by backward percolation with the static-demand evaluation function Q_{stat}.

Legend: Upgraded segment by Q_{stat} (middle blue), upgradable segments (beige), existing segments (dark gray), network (light gray)

Supp_Video_BP_Qpen.mp4

Title: Evolution of planned network over 50 years by backward percolation with the penalty-based evaluation function Q_{pen}.

Legend: Upgraded segment by Q_{pen} (dark blue), upgradable segments (beige), existing segments (dark gray), network (light gray)

Supp_Video_DO_Greedy.mp4

Title: Evolution of planned network over 50 years by greedy direct optimization.

Legend: Upgraded segment by greedy optimization (light orange), upgradable segments (beige), existing segments (dark gray), network (light gray)

Supp_Video_DO_TA.mp4

Title: Evolution of planned network over 50 years by batched direct optimization.

Legend: Upgraded segment by batched optimization (dark orange), upgradable segments (beige), existing segments (dark gray), network (light gray)
